# Probing the effect of Ni, Co and Fe doping concentrations on the antibacterial behaviors of MgO nanoparticles

**DOI:** 10.1038/s41598-022-12081-z

**Published:** 2022-05-13

**Authors:** Asma Almontasser, Azra Parveen

**Affiliations:** Department of Applied Physics, Z. H. College of Engineering & Technology, Aligarh Muslim University, Aligarh, 202002 India

**Keywords:** Biophysics, Biotechnology, Materials science, Nanoscience and technology

## Abstract

The divalent transition metal ions (Ni, Co, and Fe)-doped MgO nanoparticles were synthesized via the sol–gel method. X-ray diffraction showed the MgO pure, single cubic phase of samples at 600 °C. Field emission electron microscope showed the uniform spherical shape of samples. The magnetic behavior of Ni, Co, Fe-doped MgO system were varied with Ni, Co, Fe content (0.00, 0.01, 0.03, 0.05, 0.07). The magnetic nature of pure had changed from paramagnetic to ferromagnetic. The number of oxygen vacancies increases with increasing amounts of dopant ions that lead to an ionic charge imbalance between Ni^2+^/Co^2+^/Fe^2+^ and Mg^2+^, leading to increase magnetic properties of the samples. The magnetic nature of prepared samples makes them suitable for biomedical applications. A comparative study of the antibacterial activity of nanoparticles against the Gram-negative (*E. coli*) and Gram-positive bacteria (*S. aureus*) was performed by disc diffusion, pour plate techniques, and study surface morphology of untreated and treated bacterial cell wall. An investigation of the antibacterial activity of doped MgO nanoparticles reveals that the doped MgO nanoparticles show effective antibacterial activity against the Gram-negative (*E. coli*) and Gram-positive (*S. aureus*) bacterium. The minimum inhibitory concentration of the synthesized nanoparticles against microorganisms was recorded with 40 μg/ml, while the maximum inhibitory concentration was observed with 80 μg/ml. At a concentration of 80 μg/ml, the complete growth inhibition of the *E. coli* was achieved with 7% Co-doped MgO and 7% Fe-doped MgO, while bacterial growth of *S. aureus* was inhibited by 100% in the presence of 7% Fe-doped MgO. The present work is promising for using nanomaterials as a novel antibiotic instead of the conventional antibiotics for the treatment of infectious diseases which are caused by tested bacteria.

## Introduction

Over the past few decades, many advances have been made in the area of the preparation of nanomaterials. Many metal oxide nanostructures such as MgO, Cr_2_O_3_, In_2_O_3_, Fe_3_O_4_, TiO_2_, and ZnO are synthesized for different applications, they have attracted considerable attention from both academics and technologists due to their excellent properties and their ability to withstand harsh environments^1^. Magnesium oxide (MgO) is an important nanoscale material, and it has wide applications depending on its intrinsic properties. The size and morphology of metal oxide nanoparticles can be modified using parameters such as ionic strength, pH, different calcination temperatures, reaction temperature, and time. MgO nanoparticles can be synthesized via different techniques such as sol–gel^2,3^, hydrothermal reaction^4,5^, direct chemical transformation^6^, laser ablation^7^, aerogel^8^, microwave radiation^9^, solid-state interfacial diffusion–reaction^10^, vapor–solid process^11^, physicochemical technique^12^, chemical vapor synthesis^13^, and chemical precipitation method^14,15^, etc. Among the different synthesis methods, the sol–gel routes are a powerful, fast and economical technique for the synthesis of MgO metal oxide nanoparticles, as well as, it provides high purity homogenous materials. The sol–gel method has become a very active area within the extensive field of nanoparticle research, opening up considerable opportunities to access a broad variety of binary, ternary and doped transition metal oxide nanoparticles with high crystalline and well-defined particle morphologies. In contrast to other methods such as microwave and hydrothermal routes that are highly sensitive to the reaction conditions, sol–gel routes offer a very robust synthetic methodology to directly prepare MgO nanoparticles. Another major advantage of sol–gel routes compared to solvothermal is that the reactions are performed at lower temperatures and without the use of any special reducing agent/capping agents to prepare MgO nanoparticles. Many metal oxide nanoparticles have been developed with different crystal structures such as cubic, hexagonal, etc. Chen et al.^16^ synthesized cuboid structure of MgO nanofilm via the sol–gel method and verified its antibacterial activity by the control experiments of bacterial contamination, and also demonstrated that the cuboid structure of MgO nanofilm could withstand about 2000 °C by the refractory and high temperature resistance test. Azzam et al.^17^ synthesized MgO nanohexagon as a potential antibacterial platform agent against microorganisms in the water, and pointed that the hexagonal sheet of MgO with a high surface area promoted the ROS production, thereby causing cytotoxicity against bacterial cells. Yamamoto et al.^18^ pointed that the change of antibacterial activity of MgO–ZnO nanocubic on *E. coli* was similar to those on *S. aureus.* Mohamed et al.^19^ proved the ability of the hexagonal phase crystal of ZnO and CuO nanoparticles to inhibit several pathogens including Gram-positive and Gram-negative bacteria.

MgO nanoparticles have been primarily concerned with enhancing and developing new materials due to its fascinating optoelectronic properties, defect-induced magneto-optical properties, and broad band gap. This material can be broadly used in different fields like refractory industry, waste water treatment, remediation of toxic waste, and catalyst according to its chemical, optical, magnetic, electronic, mechanical, and thermal properties^20–29^. The foreign metal dopants in the MgO crystal lattice results in drastic changes in the properties of MgO nanoparticles. The lattice structure, grain size, band gap crystal structure, breaking and coupling of ions and atoms, and electron configuration and state might be determined utilizing the preparation pathway, which is considered a significant parameter for modifying the properties of nano-oxide materials. Consequently, the synthesis route and preparation method can change the MgO nanoparticle properties by doping a foreign element into MgO lattice depending upon the various desired applications.

MgO nanoparticles wide band gap semiconductor doped with the transition metal ions has received significant interest as they serve applications in the opto-magnetic devices and field of spintronics. MgO nanoparticles is a high hygroscopic material and easy to form diamagnetism of magnesium hydroxide_,_ reducing ferromagnetic ordering^30^. To date, no research groups have shown the ferromagnetic behavior in a MgO bulk structure experimentally. So, the magnetic properties of MgO are still unclear. Oxygen vacancy can induce the ferromagnetic behavior of MgO nanoparticles, and hydrogen adsorption can drastically enhance the magnetic moments. The origin of ferromagnetism is still debated and demands careful investigation. MgO nanoparticles is one of the most attractive model materials to examine ferromagnetism. It has a wide band-gap rock salt structure. Moreover, it is an essential barrier material used for magnetic tunnel junctions^31^. In recent years, X-ray photoelectron and photoluminescence spectroscopies indicated that the ferromagnetic behavior in the MgO system might be attributed to the Mg vacancies. Stoneham et al. reported that the magnetism induced by intrinsic defects in MgO might be correlated to the charged defects, as the experimental results explained that cation vacancy in MgO was exhibited in both charge states: singly charged ${\text{V}}_{\text{Mg }}^{-}$ and neutral ${\text{V}}_{\text{o }}^{0}$^32^. Kumar et al. pointed out that the low population of oxygen vacancies showed the diamagnetic behavior in MgO lattice and confirmed a strong relation between ferromagnetic nature, Refs.^33,34^ oxygen vacancies, and adsorbed H species^30^. The theoretical calculations and experimental results revealed that the magnetic moment of the system could be induced by cation vacancy, but the ferromagnetism of MgO nanoparticles remains debatable.

Metal oxide nanoparticles are considered one of the newest classes of materials used in biomedical applications. Magnesium oxide nanoparticles have been used in several applications such as pharmaceutical, waste remediation, glass industry, and refractory materials^35–37^. MgO nanoparticles are also used in catalyst and catalyst support applications^38–40^, etc. Many recent studies have been concerned with the antibacterial activity and the development of new and effective antibacterial reagents^41^. Due to the increasing antibiotic resistance among bacteria, the development of active antimicrobial nanomaterials sources was essential and required. The disease-causing bacteria can resist the multiple-drug because of using random drugs used for treating the infectious diseases. In addition, antibiotics sometimes have side effects such as hypersensitivity reactions, allergic reactions, and immune suppression^42^. Consequently, there must be an extensive study in the use of nanoparticles in biological and medical applications.

Herein, we utilized a sol–gel synthesis method to synthesize spherical shaped and cubic phase of Ni_,_ Co, and Fe doped-MgO nanoparticles. The structural, optical, magnetic properties of MgO nanoparticles with the amount of doping ions were observed. Moreover, the effect of doping ratio on antibacterial activity has been studied. It is perhaps noteworthy that this is the first study to demonstrate that 7% of Co and Fe-doped MgO nanoparticles with the mean diameter of 13.5 nm and 10.5 nm showed the excellent ability to completely restrict the growth of infectious bacteria.

## Materials and synthesis methods

### Chemicals

Magnesium(III) nitrate hexahydrate Mg (NO_3_)_2_·6H_2_O, Nickel(II) chloride hexahydrate (NiCl_2_·6H_2_O), Cobalt(II) nitrate Co (NO_3_)_2_, and Iron (III) nitrate nonahydrate Fe (NO_3_)_3_·9H_2_O, non-2-methyl-pyrrolidone (NMP) C_5_H_9_NO and distilled water.

### Microorganisms

The effects of antibacterial of MgO metal oxide nanoparticles and transition metal (Ni, Co, and Fe)-doped MgO metal oxide nanoparticles were assessed against *E. coli and S. aureus*.

### Preparation of Ni-doped MgO nanoparticles

**Figure 1:**
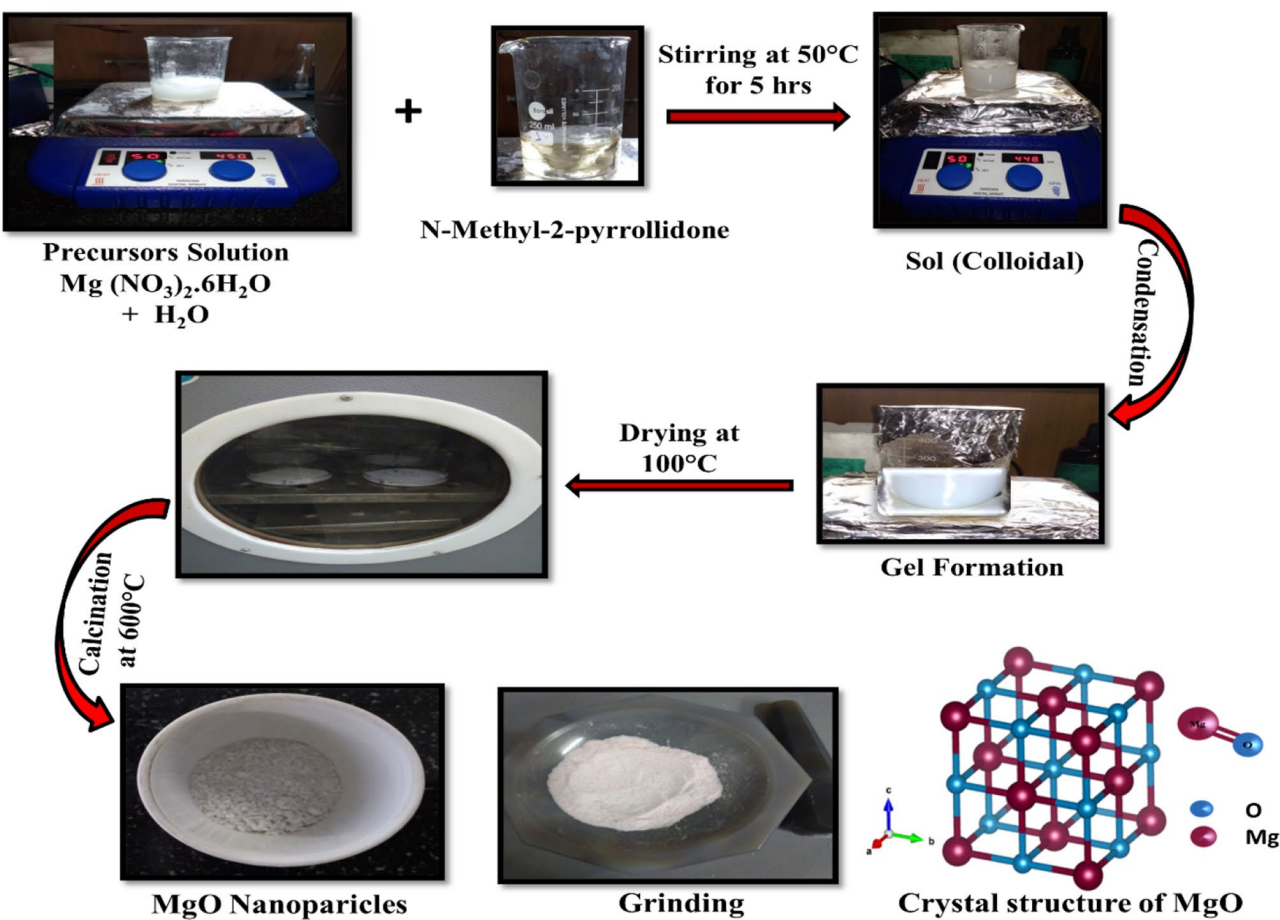
Schematic illustration showing the formation mechanisms of MgO nanoparticles via the sol–gel process.

Ni-doped MgO nanoparticles were synthesized successfully by sol–gel methods with the different concentrations of Ni ions at room temperature under atmospheric condition. A mixture of Mg (NO_3_)_2_·6H_2_O (7 g) and appropriate amounts of NiCl_2_·6H_2_O (0.5 M), distilled water (100 ml), non-2-methyl-pyrrolidone (40 ml) were stirred constantly for 5 h in the closed container at 50 °C. A viscous gel was formed, and then was centrifuged at 5000 rpm. The gel product was heated at 100 °C in an oven for 72 h. All the samples were ground and annealed in the furnace at 600 °C for 4 h. Then it was allowed to be cooled to ambient temperature over, and giving ultrafine powder as a product. The obtained product of Ni-doped MgO nanoparticles had varying shades of gray color ranging from light to dark which varied according to the increase of the amount of Ni dopant. The formation mechanisms of MgO nanoparticles by the sol–gel method are illustrated in Fig. 1.

### Preparation of Co-doped MgO nanoparticles

Co-doped MgO nanoparticles were synthesized via the sol–gel process with the different concentrations of Co ions at low temperature. A mixture of Mg (NO_3_)_2_·6H_2_O (7 g), Co (NO_3_)_2_·6H_2_O (0.5 M), distilled water (100 ml), and non-2-methyl-pyrrolidone (40 m) was stirred for 5 h at 50 °C continuously to mix the solution uniformly. After evaporating all the water, a viscous gel was formed and centrifuged at 5000 rpm. The gel product was heated at 100 °C in an oven for 72 h. All the samples were ground and annealed in the furnace at 600 °C for 4 h. Then it was allowed to be cooled to ambient temperature and obtained ultrafine powder. The ultrafine powder collected had various shades of gray color that varied from bright to deep-gray due to the increased Co dopant.

### Preparation of Fe doped MgO nanoparticles

Fe-doped MgO nanoparticles have been prepared by the sol–gel technique with the different concentrations of Fe ions. Mg (NO_3_)_2_·6H_2_O (7 g), Iron nitrate nonahydrate Fe (NO_3_)_3_·9H_2_O, non-2-methyl-pyrrolidone (40 ml), distilled water (100 ml), and non-2-methyl-pyrrolidone (40 ml) was stirred for 5 h at 50 °C continuously to mix the solution uniformly. After evaporating all the water, a viscous gel was formed. The gel was washed several times with distilled water and ethanol using centrifuging technique at a rate of 5000 rpm. The gel product was heated at 100 °C in an oven for 72 h. All the samples were placed into the furnace at 600 °C for 4 h. Then it was allowed to be cooled to ambient temperature. The obtained product of Fe-doped MgO nanoparticles had varying shades of brown color, which diverse according to the amount of Fe dopant from bright-brown to deep-brown.

## Results and discussion

### Characterization

Structure and surface morphology as-synthesized of Ni, Co, and Fe-doped MgO nanoparticles were analyzed by x-ray diffraction (XRD), using CuKα radiation (λ = 1.5406 Å) and covering 2θ between 20° and 80° and FE-SEM measurements. The average diameter (D) of synthesized metal oxide nanoparticles was calculated from the broadening of the XRD peak intensity by using the Debye–Scherrer equation. The optical properties of metal oxide powders were examined by a UV–Visible spectrophotometer in the 200–900 nm wavelength range. Fourier transform infrared (FT-IR) spectroscopy was used to analyze the chemical composition of metal oxide particles, where infrared light was used to scan samples and observe the chemical properties. The infrared spectra were obtained at room temperature in the range of 4000 to 400 cm^−1^. Photoluminescence spectrophotometer characterized the spectrum of Ni, Co, and Fe-doped MgO nanoparticles. The antibacterial action of transition metal oxide nanoparticles has been studied with *E. coli* (Gram-negative bacteria) and *S. aureus* (Gram-positive bacteria). The antibacterial activity of Ni, Co, and Fe-doped MgO nanoparticles has been tested by disc diffusion test and pour plate method. This test was examined using nutrient agar (solid medium) and nutrient broth (liquid medium).

### X-ray diffraction study

**Figure 2:**
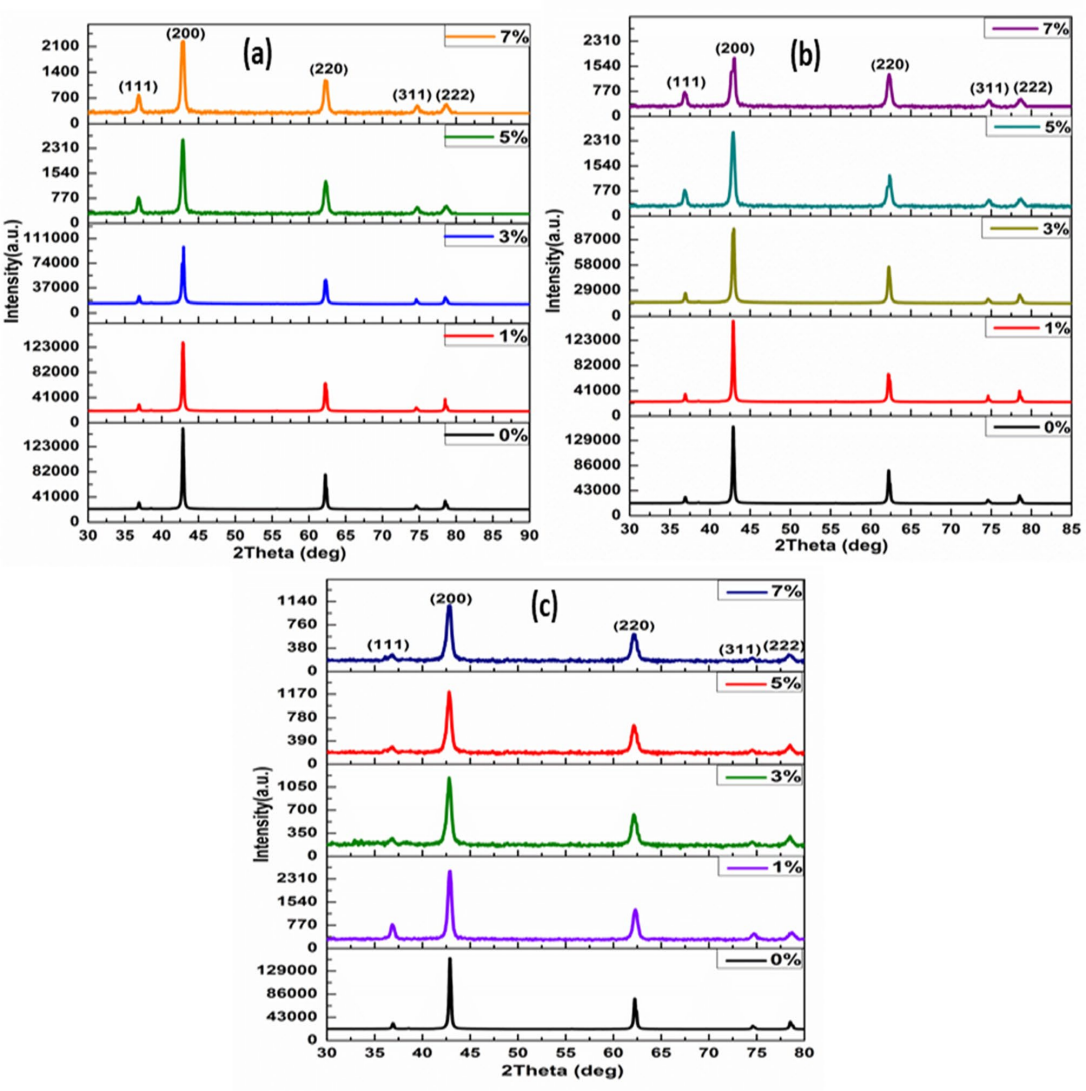
XRD of MgO pure and MgO doped with (**a**) Ni, (**b**) Co and (**c**) Fe for different concentrations (0%, 1%, 3%, 5%, and 7%) nanoparticles.

**Figure 3:**
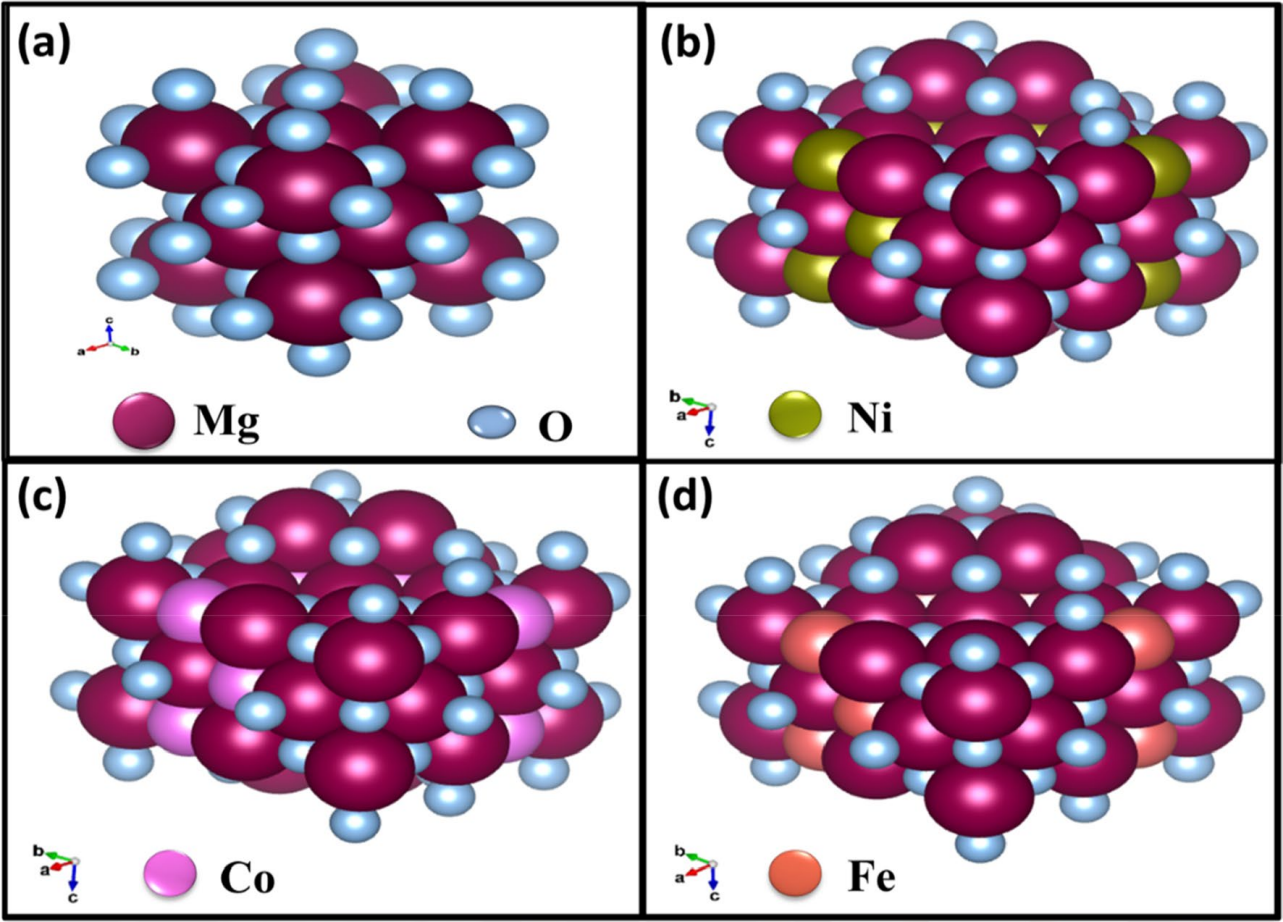
Cubic structure of (**a**) MgO, (**b**) Ni-doped MgO, (**c**) Co-doped MgO, (**d**) Fe-doped MgO nanoparticles.

Powder X-ray diffraction (PXRD) patterns of Ni_x_Mg_1−x_O, Co_x_Mg_1−x_O, and Fe_x_Mg_1−x_O nanoparticles was shown in Fig. 2a–c. The X-ray diffraction (XRD) patterns of the nanoparticles and classified samples were given by X-ray diffractometer with CuKα radiation (1.5406 Å wavelength) under voltage of 30 kV and a current of 15 mA. The as-formed samples prepared by sol–gel technique showed pure single cubic phase, and no secondary phase was detected with calcination at 600 °C. The cubic structure of the prepared samples was depicted in Fig. 3a–d. The XRD patterns of samples confirmed that no detectable contamination in the particles has occurred. XRD pattern of all synthesized samples was indexed by Powder X software. We have observed a slight shift (blue shift) in diffraction peak towards the higher diffraction angle on substitution of (Ni, Co, and Fe) ions in the magnesium oxide lattice as presented in Fig. 2a–c. All the X-ray diffraction peaks of Ni, Co, and Fe)-doped MgO nanoparticles at 2θ assigned to (1 1 1), (2 0 0), (2 2 0), (3 1 1), and (2 2 2) planes of the prepared samples^43^, and indexed well to the cubic structure of the Ni, Co, and Fe-doped MgO nanoparticles^44^. The lattice parameter of synthesized magnesium oxides was a = 4.21 Å, in good agreement with JCPDS PDF data (no. 45-946). The crystallite size of MgO metal oxide and transition metal (Ni, Co, and Fe)-doped MgO nanoparticles were determined from the broadening full-width at the half-maximum (FWHM) of the X-ray the diffraction peaks using Scherrer’s Eq. (1)^45^:

1 $$D=\frac{K\lambda }{\beta {\text{cos}}\theta },$$

where D is the crystalline size, *K* is a constant, which is equal to 0.94, λ is the wavelength of CuKα radiation (1.5406 Å), β is the width at the half maximum of the peak (2 0 0), θ is the diffraction Bragg angle.

The XRD peaks were shifted slightly to a higher angle side with an increased doping concentration of Ni, Co, and Fe. Also, we observed that the crystallite size was decreased with an increase of Ni, Co, and Fe concentrations. The decrease in the mean size of the nanocrystals indicates that Ni, Co, and Fe ions doped into the host lattice were strongly affected on the crystal lattice.

The crystallite peak width is interconnected to the crystallite size. The width of the peak varies universally with the crystallite size. In the X-ray diffraction pattern, the smaller crystallites sizes promote broader peaks in the crystal plane, the higher intensities of the crystalline peaks indicate the larger crystallite size^46,47^.

The average crystallite size of the MgO metal oxide nanoparticles was evaluated using Scherrer’s formula to be ∼ 14.71 nm. The average crystallite size of Ni-doped MgO nanoparticles ranged from 14.15 to 12.43 nm in diameter, while the mean crystallite size of Co-doped MgO nanoparticles was in the range from 14.51 to 11.49 nm. The average crystallite size of Fe-doped MgO nanoparticles ranged from 13.88 to 10.57 nm. All the results of the average crystallite size of Ni, Co and Fe-doped MgO nanoparticles for different doping concentrations were tabulated in Table 1.

**Table 1:** Specimen name, the average crystallite size (D) of the specimens, and energy band gab.

Specimen name	Crystallite size (D) (nm)	Energy band gab (eV)
MgO	14.71	5.45
Ni_0.01_ Mg_0.99_O	14.21	5.61
Ni_0.03_ Mg_0.97_O	13.89	5.77
Ni_0.05_ Mg_0.95_O	13.55	5.94
Ni_0.07_ Mg_0.93_O	12.43	6.23
Co_0.01_ Mg_0.99_O	14.15	5.62
Co_0.03_ Mg_0.98_O	13.02	5.84
Co_0.05_ Mg_0.95_O	12.68	6.06
Co_0.07_ Mg_0.93_O	11.49	6.31
Fe_0.01_ Mg_0.99_O	13.88	5.71
Fe_0.03_ Mg_0.98_O	12.64	6.07
Fe_0.05_ Mg_0.95_O	11.90	6.27
Fe_0.07_ Mg_1.93_O	10.75	6.57

From Table 1, we observed that increasing the concentration of Ni, Co, and Fe reduced the size of the nanoparticles of prepared samples. The results provide evidence that the ions of Ni, Co, and Fe were successfully substituted into the structure of the MgO matrix^48^. The ionic radius of (Ni^2+^_,_ CO^2+^_,_ and Fe^2+^) was close to the ionic radius of Mg^2+^, making it likely that (Ni^2+^_,_ CO^2+^_,_ and Fe^2+^) ions would substitute with Mg^2+^ in MgO lattice^49^. XRD analysis demonstrated that dopants (Ni^2+^_,_ CO^2+^_,_ and Fe^2+^) were tightly substituted into the Mg^2+^ sites of the MgO host lattice, which was possible since the ionic radius of Ni^2+^ (0.069 nm), CO^2+^ (0.065 nm), and Fe^2+^ (0.064 nm) were smaller than the ionic radius of Mg^2+^ (0.072 nm). The reduction in the crystallite size was mainly distortion in the host lattice MgO by the hosted ions (Ni^2+^, CO^2+^, and Fe^2+^), leading to a decrease in the nucleation and inhibiting the growth of MgO nanoparticles.

The average crystallite size values of the synthesized samples were listed in Table 1, which decreases with increasing the transition metal (Ni, Co, and Fe) dopant concentrations. The lattice size was reduced by doping, especially when the doping happened with larger atoms.

**Figure 4:**
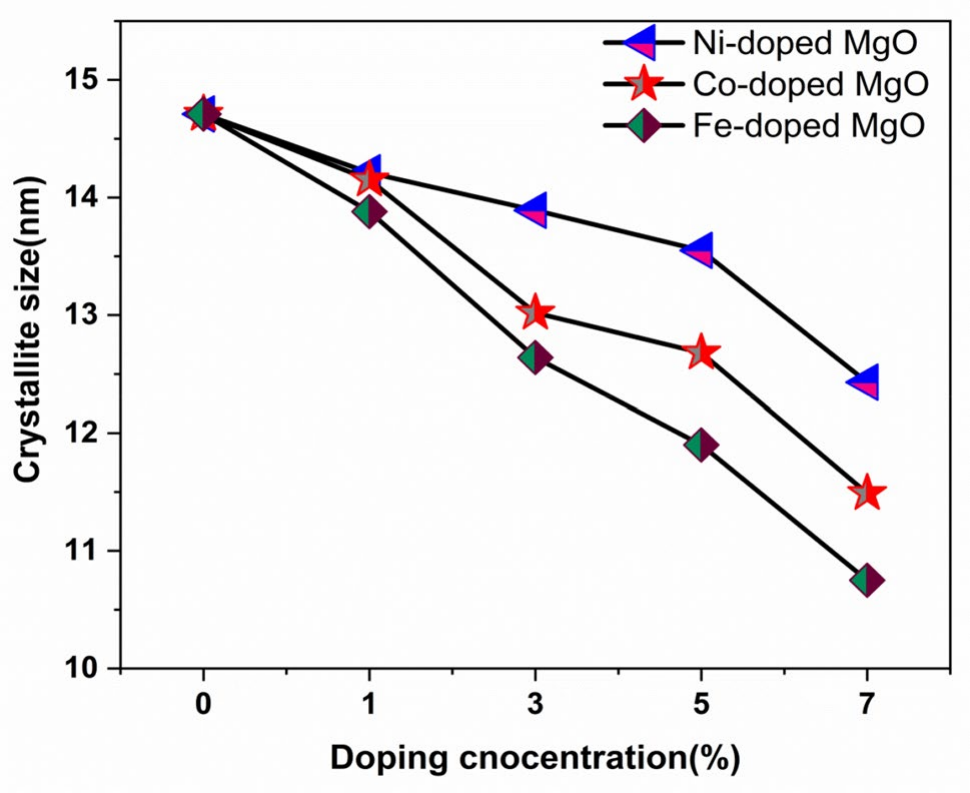
The correlation between the doping ratio and crystallite size of divalent metal ions (Ni, Co, and Fe) doped MgO nanoparticles.

The crystallite size and dopant concentrations were tabulated for all the prepared samples. These values were plotted in Fig. 4 to depict the correlation between Ni, Co, and Fe concentrations and crystallite size. Crystallite size of Ni, Co, and Fe-doped MgO nanoparticles decreased with increasing the transition metal (Ni, Co, and Fe) dopant concentrations.

### Field-emission scanning electron microscope (FE-SEM)

**Figure 5:**
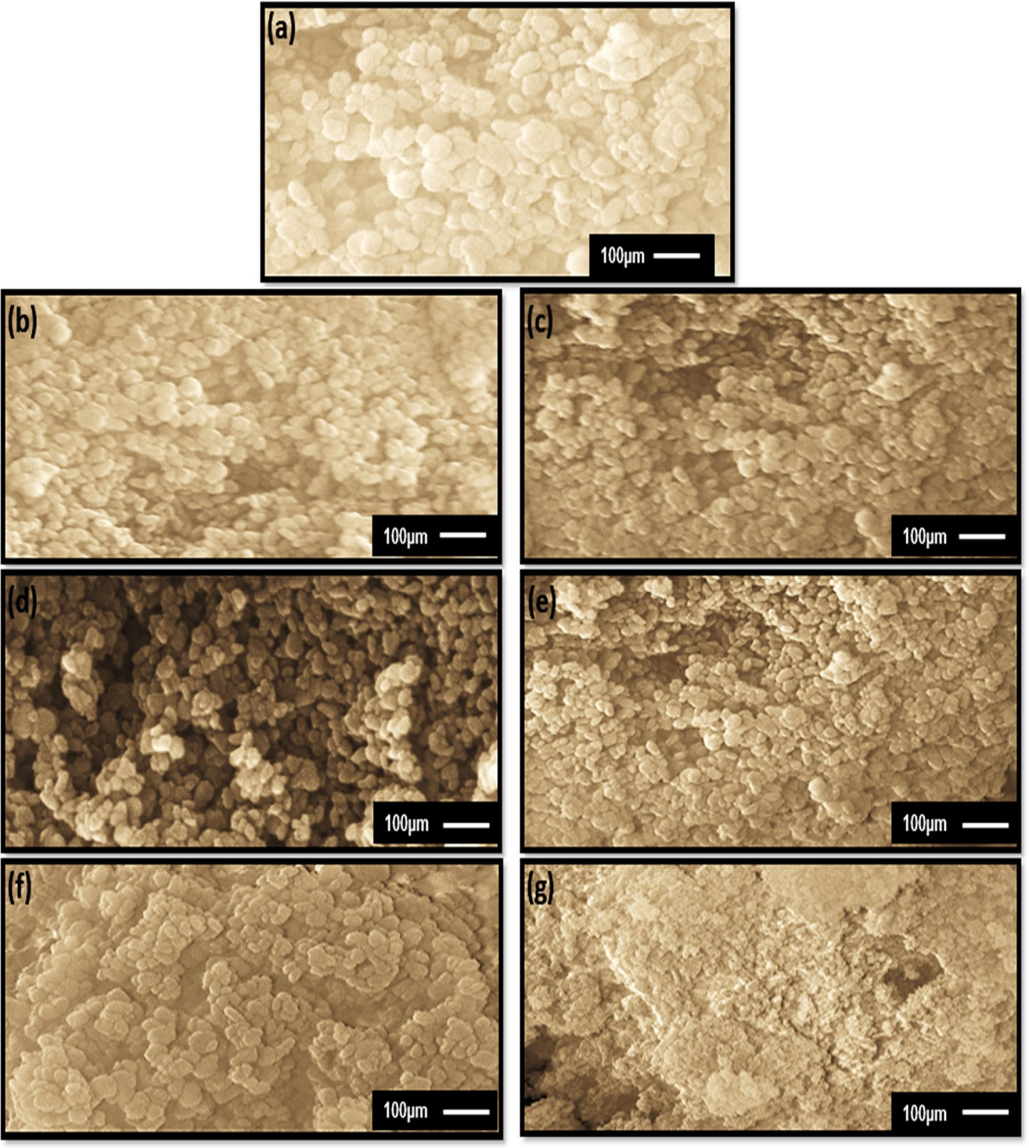
FE-SEM microstructures of (**a**) Pure MgO nanoparticles, and MgO nanoparticles doped with 3%, 7% of (**b,c**) Ni, (**d,e**) Co, and (**f,g**) Fe.

**Figure 6:**
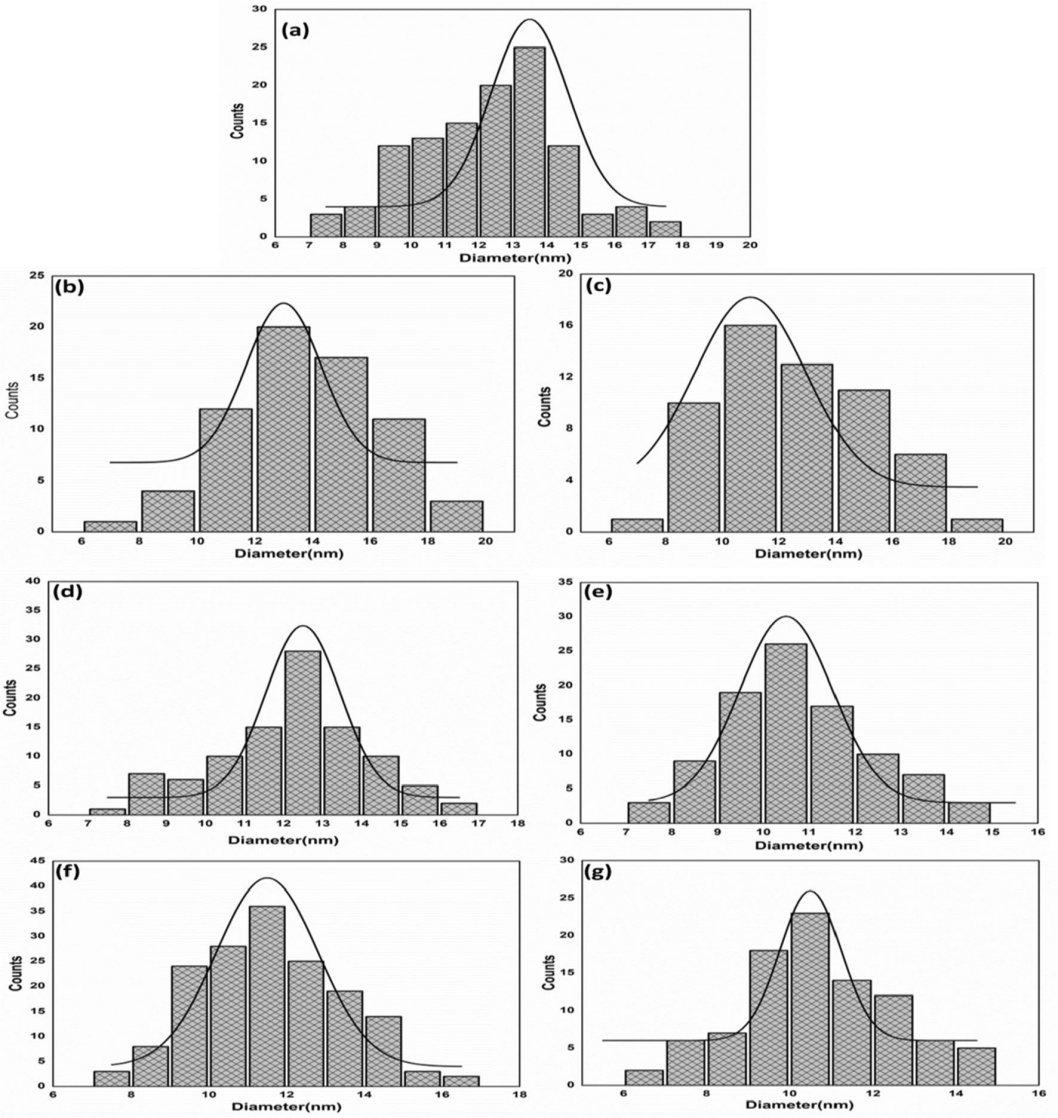
Particle size distribution curves of (**a**) MgO and MgO nanoparticles doped with 3%, 7% of (**b,c**) Ni (**d,e**) Co, and (**f,g**) Fe.

The FE-SEM inspection of samples showed changes in the size of particles as a function of concentrations of doping ions. Figure 5a–g displays the morphology and statistical particle size distribution of Ni, Co and Fe-doped MgO. FE-SEM microstructure of samples shows homogeneous and uniform distribution of particles in a spherical-shape manner^50^. These particles are highly monodispersed and of narrow size distribution. It is also noted that all particles get agglomerated on their surface. Agglomeration of particles on the surface might have originated from the high surface energy of the synthesized nanoparticles. This is a direct result of the synthesis route applied in the present work. Figure 6a–g shows the mathematical model (Gaussian curvature) to determine the maximum probability of particle size distribution. The particles distribution in all samples cases were uniform, and their particle size was nearly equal to particle size in XRD data. Figure 6a illustrated that the particle size of MgO was found to be in the range between 7 and 18 nm with an average diameter of 13.5 nm. For 3% and 7% of Ni-doped MgO nanoparticles, the particles size was in the range of 6–20 nm with the mean particle diameter of 13 nm and 11 nm (see Fig. 6b,c). The average diameter of particles for (3% and 7%) Co-doped MgO was equal to 12.5 nm and 10.5 nm, respectively (Fig. 6d,e). 7–17 nm was the particle size for 3% of Co-doped MgO nanoparticles and 7% of Co-doped MgO particles ranged between 7 and 15 nm. Transition metal 3% Fe-doped MgO nanoparticles have particles size lies between 7 and 17 nm. In contrast, transition metals of 7% Fe-doped MgO nanoparticles have sizes ranging between 6 and 15 nm. Figure 6f,g showed that the values of 11.5 nm and 10.5 nm were the average diameters of particles for (3%, and 7%) Fe-doped MgO nanoparticles, respectively. The results of the FE-SEM inspection corroborated the conclusions based on the results of XRD measurements. A decrease in crystallite size was influenced by adding transition metal dopants (Ni, Co, and Fe) content into MgO host lattice.

### UV–Visible spectrophotometer and Tauc’s plot

**Figure 7:**
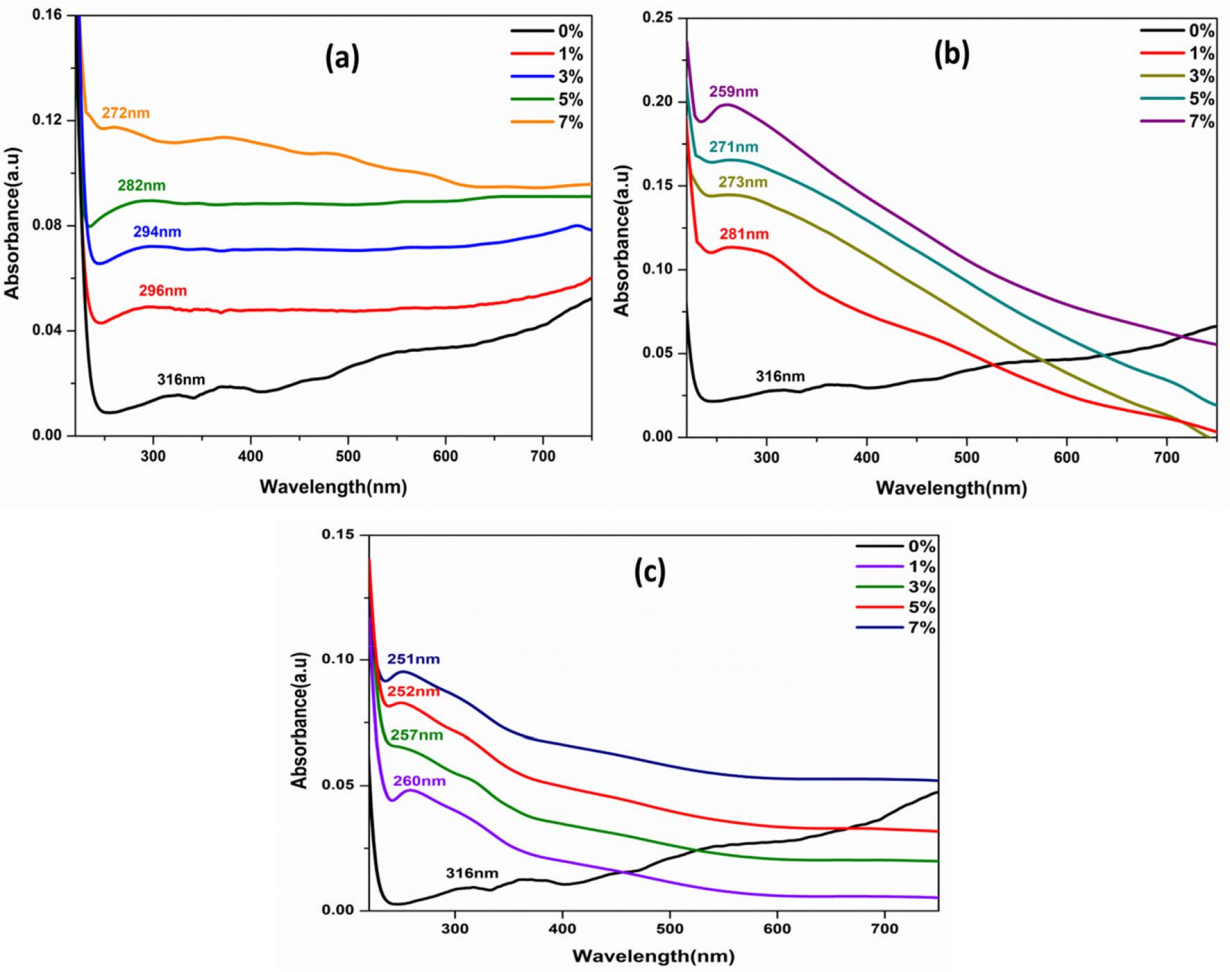
UV–Visible absorption spectra of MgO nanoparticles doped with (**a**) Ni, (**b**) Co and (**c**) Fe at different concentrations (0%, 1%, 3%, 5%, and 7%).

The optical absorbance spectrum of the synthesized samples was analyzed at room temperature using a UV–Vis spectrophotometer. Figure 7a–c shows the UV–Visible spectrum of the Ni, Co, and Fe-doped MgO nanoparticles, recorded in the 200–750 nm wavelength region. In the main, the different factors such as band gap, lattice strain, impurity centers, grain size, surface roughness, and oxygen deficiency are related to the absorbance spectrum of the materials. From Fig. 7a–c, the absorption edge of transition metal (Ni, Co, and Fe)-doped MgO nanoparticles shifted toward a lower wavelength than the absorption edge of MgO metal oxide. Among them, the absorption spectrum of transition metal Fe-doped MgO nanoparticles shifted toward a lower wavelength than the absorption spectrum of transition metal Ni and Co-doped MgO nanoparticles. Thus, the blue shift in the absorption spectrum of all samples appeared with increasing the doping concentrations of the transition metal Ni, Co, and Fe, as observed in Fig. 7. Thus, the blue shift of the absorption spectrum of the prepared samples was conceivable. In contrast, the transition metal ions of (Ni^2+^, Co^2+^, and Fe^2+^) were incorporated totally into the Mg^2+^ sites of the MgO host lattice^51^.

**Figure 8:**
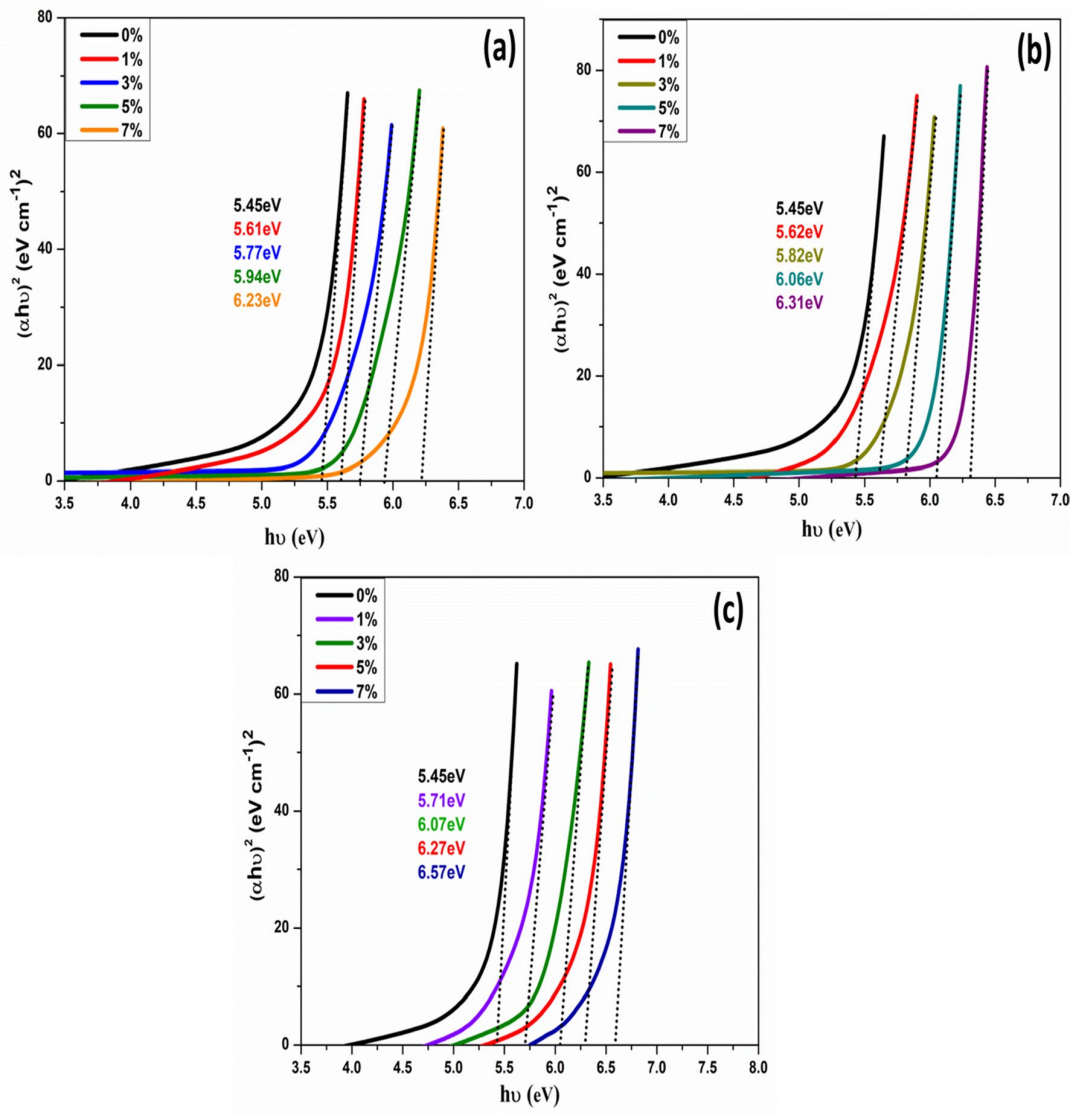
Variations of (αhν)^2^ with (hν) for Pure MgO metal oxide and transition metal MgO NPs doped with: (**a**) Ni, (**b**) Co and (**c**) Fe with the different concentrations at room temperature.

The energy band gap of the samples is estimated by Tauc’s plot Fig. 8a–c using the following Tauc’s Eq. (2)^52,53^.

2 $$(\alpha h\nu ) = A \, (h\nu {-}E_{g} )^{r} .$$

α is the optical absorption coefficient of the materials, hν is the photon energy, E_g_ is the direct band gap, A is a constant, and r is equal to ½ for direct allowed transitions. In order to determine the optical band-gap values, the variation of the factor (αhν)^2^ as a function of the incident photon energy (hν) was plotted in Fig. 8a–c, The intercept of the extrapolated straight-line portion of the curves to zero absorption coefficient value gives the energy band gap value. Every value of energy band gap for transition metal (Ni, Co, and Fe)-doped MgO nanoparticles with different concentrations was tabulated in Table 1.

From Tauc’s plot, the energy band gap of pure MgO nanoparticles was about 5.45 eV. The energy band gap of pure MgO nanoparticles (5.45 eV) was smaller than the energy band gap of bulk MgO (7.8 eV). The difference in band gap energy of NPs with their bulk materials may be attributed to planar defects. On the other hand, other researchers recorded that the band gap value for MgO nanoparticles was smaller than the obtained band gap value by this work^54^. This behavior might ascribe to the quantum confinement in which the optical band gap of the manufactured materials increases with decreasing nanoparticles.

The nanomaterials with a quantum dot radius show size-dependent optical properties. This behavior results from quantum confinement effects (QCE) of the charge carrier (hole–electron)^55^. So, the confinement occurs, leading to the transition from continuous to discrete energy levels.

Generally, the size of nanoparticles could create a change in the band gap energies of the materials. The particle size of MgO nanoparticles doped with Ni, Co, and Fe were decreased with the increment of dopant concentrations, as depicted in Fig. 8a–c. The lattice size of samples was reduced due to the radius of doping transition ions, that it was possible to occur due to the ionic radius of Ni^2+^ (0.069 nm), Co^2+^ (0.065 nm), and Fe^2+^ (0.064 nm) were smaller than the ionic radius of Mg^2+^ (0.072 nm). These results signified that transition metal ions of Ni^2+^, Co^2+^, and Fe^2+^ have been incorporated successfully into MgO nanoparticles by substituting Mg^2+^ sites of the lattice. Moreover, the band gap energies of the used nanoparticles increased with the average crystallite size decrease. The confined dimension decreased according to the reduced particle size of the samples. The reduction in confinement dimension yields discrete energy levels of materials, whereas the band gap broadens up, ultimately resulting in increasing gap energy. As depicted in Fig. 8a–c, the energy band gap of transition metal Fe-doped MgO nanoparticles was higher than the energy band-gap of the transition metal (Ni and Co)-doped MgO nanoparticles. This result might be due to the smaller particle size of transition metal Fe-doped MgO nanoparticles as compared to the particle size of the transition metal (Ni, and Co)-doped MgO nanoparticles as recorded in Table 1.

### Photoluminescence measurements

**Figure 9:**
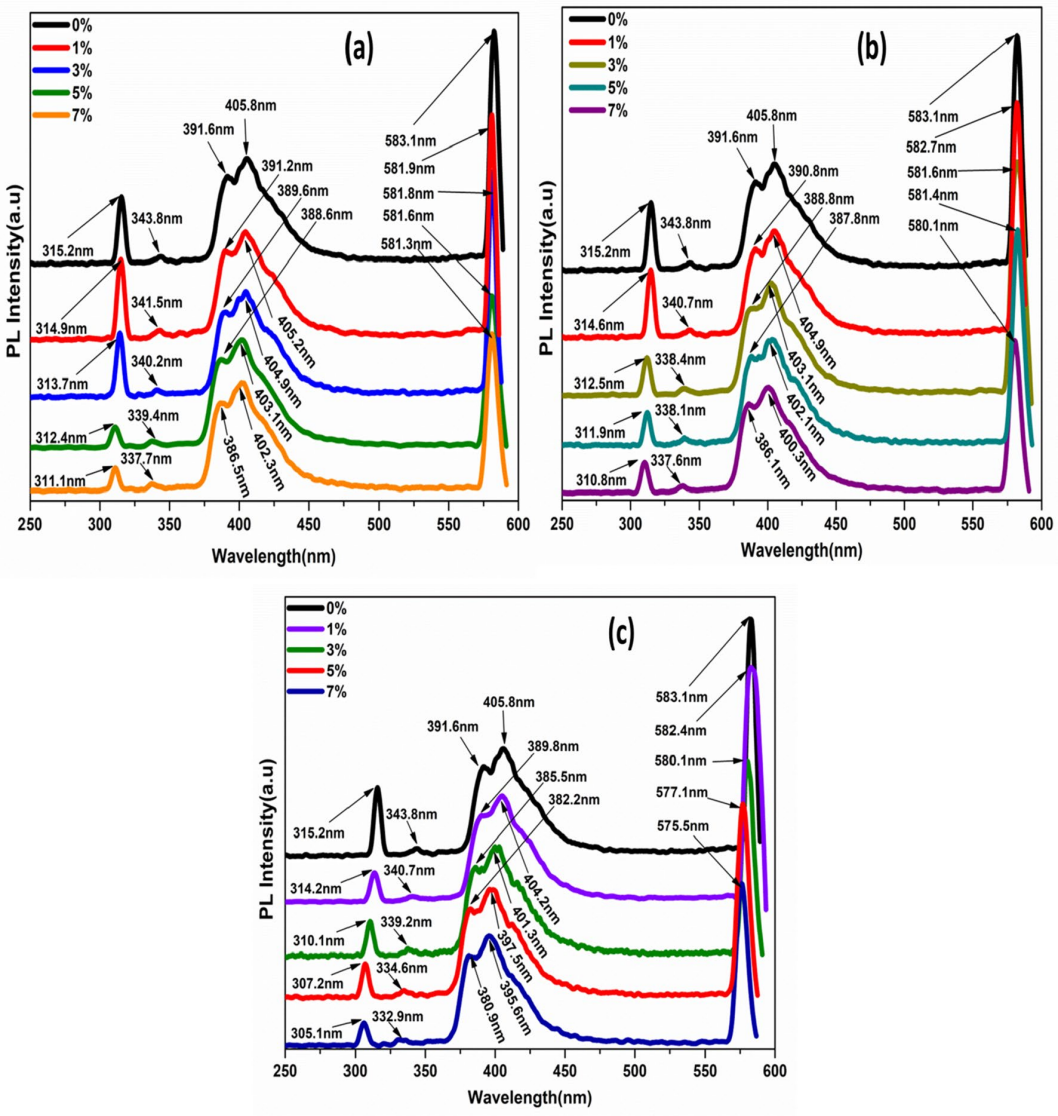
Photoluminescence spectra of Pure MgO metal oxide and transition metal MgO nanoparticles doped with: (**a**) Ni, (**b**) Co and (**c**) Fe with the different concentrations.

PL spectra of MgO nanoparticles doped with Ni, Co, and Fe were illustrated in Fig. 9a–c. Generally, PL emission was ascribed to the existence of vacancies (magnesium or oxygen) or maybe the presence of defects (interstitial magnesium or anti-site oxygen). The presence of vacancies in the nanostructures is responsible for creating a new energy level within the band gap, leading to the emissions generated from their trap levels. While exciting materials, the emissions arise due to radiative recombination of photoexcited electrons and holes. Due to oxygen vacancies, the emission peaks are indicated as trap state or deep level emissions.

Figure 9a–c illustrated the Ni, Co, and Fe-doped MgO nanoparticles and cleared that the peaks of all samples were shifted toward the smaller wavelength. The blue shift for all samples occurred as the atomic percentage of metal doping increased due to the size quantization. With increasing the transition metal concentrations from 1 to 7%, the intensity of the emission band increased. This possible occurrence is due to decreasing particle size and thus increasing surface area upon the doping percentage compared to the pure MgO nanoparticles. The doping with metal ions of Ni, Co, and Fe smaller size than Mg ion signified more structural defects than the pure MgO nanoparticles due to the crystal lattice contraction^56^.

In the photoluminescence spectra of all prepared samples, five emission peaks are observed in Fig. 9a–c. The excitation wavelength of pure MgO nanoparticles was observed at 307 nm. Two emission peaks of pure MgO nanoparticles centered at 315.2 nm, and 343.8 nm are ascribed as ultraviolet emission, i.e., near band-edge (NBE) emission. The ultraviolet emission is related to magnesium vacancies where the energy interval between the bottom of the conduction band and the magnesium vacancy (V_Mg_) level are 6.31 and 5.78 eV. Violet emission band positioned at 391.6 and 405.8 nm, the violet emission probably attributed to radiative defects correlated to trapping states existing at grain boundaries. So, the emission peaks at 391.6 and 405.8 nm appeared due to radiative transition between the valence band and trapping level. A yellow emission region peaked at 583.1 nm, and this emission was created due to the recombination of electrons with holes trapped in singly ionized oxygen vacancies.

The PL spectra of transition metal 1% Ni-doped MgO nanoparticles have five peaks originating around 314.9, 341.5, 391.2, 405.2, and 581.9 nm. The first and second peak corresponds with the ultraviolet region. The other three peaks correlate to violet, violet, and yellow in the visible region, respectively. The intensity of emission peaks varies as the concentration of Ni in the MgO nanoparticles varies, as shown in Fig. 9a. Furthermore, the peak was shifted toward a lower wavelength with increasing Ni ion concentrations.

Figure 9b presented photoluminescence (PL) spectra at room temperature of transition metal Co-doped MgO nanoparticles at different concentrations. Five peaks were observed in the photoluminescence spectra. In the transition metal, 1% Co-doped MgO nanoparticles the near band edge (NBE) emission observed at 314.6 and 340.7 nm (ultraviolet emission). Two peaks appeared at about 390.8 and 404.9 nm, corresponding to a violet emission band. Finally, the yellow emission band was observed at 582.7 nm in the visible region.

Figure 9c depicted photoluminescence (PL) spectra of transition metal Fe-doped MgO nanoparticles with different doping concentrations. Five peaks were exhibited for all the different concentrations of the transition metal Fe-doped MgO nanoparticles. For example, on the spectra of transition metal 1% Fe-doped MgO nanoparticles, there are two peaks centered at around 314.2 and 340.7 nm in the ultraviolet (UV) region attributed to the near band edge (NBE) emission. Violet emission peaks were centered at 389.8 and 404.2 nm, and yellow emission peaks appeared at 582. 4 nm. The peak intensities varied with the increasing ion doping concentrations for all prepared samples, as observed in Fig. 9c. The peak, wavelength, and energy band gap values of pure and different transition metal oxide concentrations-doped MgO nanoparticles are tabulated in Table 2.

**Table 2:** The peak, wavelength, and energy band gap values of pure and different transition metal oxide concentrations-doped MgO nanoparticles.

Sample name	Peak	λ (nm)	E (eV)
MgO	UV	315.2	6.3065
	UV	343.8	5.7819
	Violet	391.6	5.0762
	Violet	405.8	4.8985
	Yellow	583.1	3.4091
Ni_0.01_ Mg_0.99_O	UV	314.9	6.3126
	UV	341.5	5.8209
	Violet	391.2	5.0813
	Violet	405.2	4.9058
	Yellow	581.9	3.4161
Ni_0.03_ Mg_0.98_O	UV	313.7	6.3367
	UV	340.2	5.8431
	Violet	389.6	5.1022
	Violet	404.9	4.9094
	Yellow	581.8	3.4167
Ni_0.05_ Mg_0.95_O	UV	312.4	6.3631
	UV	339.4	5.8569
	Violet	388.6	5.1153
	Violet	403.1	4.9313
	Yellow	581.6	3.4179
Ni_0.07_ Mg_0.93_O	UV	311.1	6.3897
	UV	337.7	5.8864
	Violet	386.5	5.1431
	Violet	402.3	4.9411
	Yellow	581.3	3.4196
Co_0.01_ Mg_0.99_O	UV	314.6	6.3186
	UV	340.7	5.8345
	Violet	390.8	5.0865
	Violet	404.9	4.9094
	Yellow	582.7	3.4114
Co_0.03_ Mg_0.98_O	UV	312.5	6.361
	UV	338.4	5.8742
	Violet	388.8	5.1127
	Violet	403.1	4.9313
	Yellow	581.6	3.4179
Co_0.05_ Mg_0.95_O	UV	311.9	6.3733
	UV	338.1	5.8794
	Violet	387.8	5.1259
	Violet	402.1	4.9436
	Yellow	581.4	3.419
Co_0.07_ Mg_0.93_O	UV	310.8	6.3958
	UV	337.6	5.8881
	Violet	386.1	5.1485
	Violet	400.3	4.9658
	Yellow	580.1	3.4267
Fe_0.01_ Mg_0.99_O	UV	314.2	6.3266
	UV	340.7	5.8345
	Violet	389.8	5.0996
	Violet	404.2	4.9179
	Yellow	582.4	3.4132
Fe_0.03_ Mg_0.98_O	UV	310.1	6.4103
	UV	339.2	5.8603
	Violet	385.5	5.1565
	Violet	401.3	4.9535
	Yellow	580.1	3.4267
Fe_0.05_ Mg_0.95_O	UV	307.2	6.4708
	UV	334.6	5.9409
	Violet	382.2	5.201
	Violet	397.5	5.0008
	Green-Yellow	577.1	3.4445
Fe_0.07_ Mg_0.93_O	UV	305.1	6.5153
	UV	332.9	5.9712
	Violet	380.9	5.2188
	Violet	395.6	5.0248
	Green	575.5	3.4541

In the PL spectra of synthesized samples, the emission peaks are shown blue shift due to the quantum size effect according to the quantum confinement by varying the quantum dot size. Thus, the band gap of nanocrystals increases with decreasing nanocrystal size. These results were in good agreement with XRD results.

### Infrared spectral (FT-IR) study

**Figure 10:**
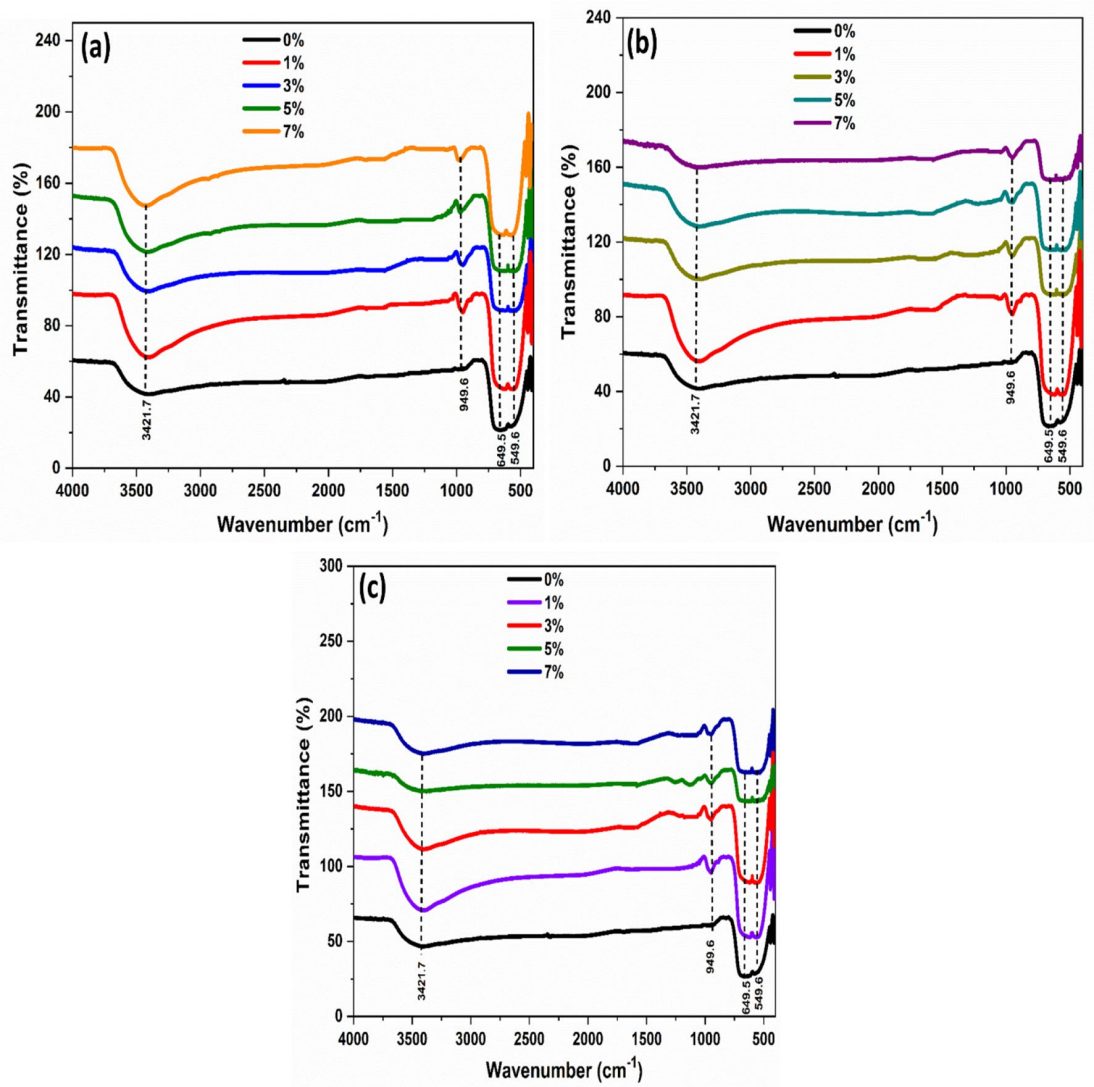
FT-IR spectra of Pure MgO metal oxide and transition metal MgO nanoparticles doped with: (**a**) Ni, (**b**) Co and (**c**) Fe with the different concentrations.

FT-IR spectroscopy was studied to identify the functional groups in the MgO metal oxide nanoparticles and transition metal (Ni, Co, and Fe)-doped MgO nanoparticles. Figure 10a–c illustrates the FT-IR spectra of all prepared samples. This clearly showed a broad absorption band of MgO metal oxide nanoparticles at 3000–3700 cm^−1^ with the absorption peak of 3421.7 cm^−1^, attributed to the O–H stretching vibration of water^57,58^. The peak present at 1622.9 cm^−1^ in spectra of MgO nanoparticles is assigned to the carboxyl groups C=O. The peaks localized at 1254.4 and 1117.2 cm^−1^ are attributed to stretching vibration carboxyl groups C–O^59^. The peak at 949.6 cm^−1^ is related to alcohol groups C–H^60^. The strong band at 649.5 and 549.6 cm^−1^ was related to the characteristic stretching vibration mode of symmetric Mg–O^61^.

The FT-IR spectra of transition metal Ni-doped MgO nanoparticles depicted its functional groups in Fig. 10a at different concentrations. The broad absorption band around 3000–3700 cm^−1^, which ws centered at ~ 3437.7 cm^−1^ was related to hydroxyl groups O–H mode. The peak appeared about 954.3 cm^−1^ ascribed to the stretching mode of (Mg, Ni)–O and Ni–O vibration mode. The peaks observed around 652.7 and 552.2 cm^−1^ were related to the Mg–O vibration modes.

Figure 10b illustrated FTIR spectra of transition metal Co-doped MgO nanoparticles with different doping concentrations. The large absorption band around 3000–3700 cm^−1^, centered at 3442.5 cm^−1^, indicated the presence of hydroxyl groups O–H of molecular water^62^. The peak localized at ~ 1635.2 cm^−1^ was related to the stretching mode of (Mg, Co)–O and Co–O vibration mode. The peak of about 956.1 cm^−1^ was related to the alcohol groups C–H. The peaks observed at low frequencies 654.7 and 555.2 cm^−1^ attributed to the Mg–O stretching vibration modes.

The functional groups of transition metal Fe-doped MgO nanoparticles are shown in Fig. 10c. The absorption peak at ~ 3445 cm^−1^ could be due to the formation of hydroxyl groups O–H mode of water. The stretching mode of (Mg, Fe)–O and Fe–O vibration mode centered at 959.4 cm^−1^. At low frequencies of 665.5 and 567.4 cm^−1^, a sharp band appeared corresponding to stretching vibrations of Mg–O bonding.

### Magnetic properties

**Figure 11:**
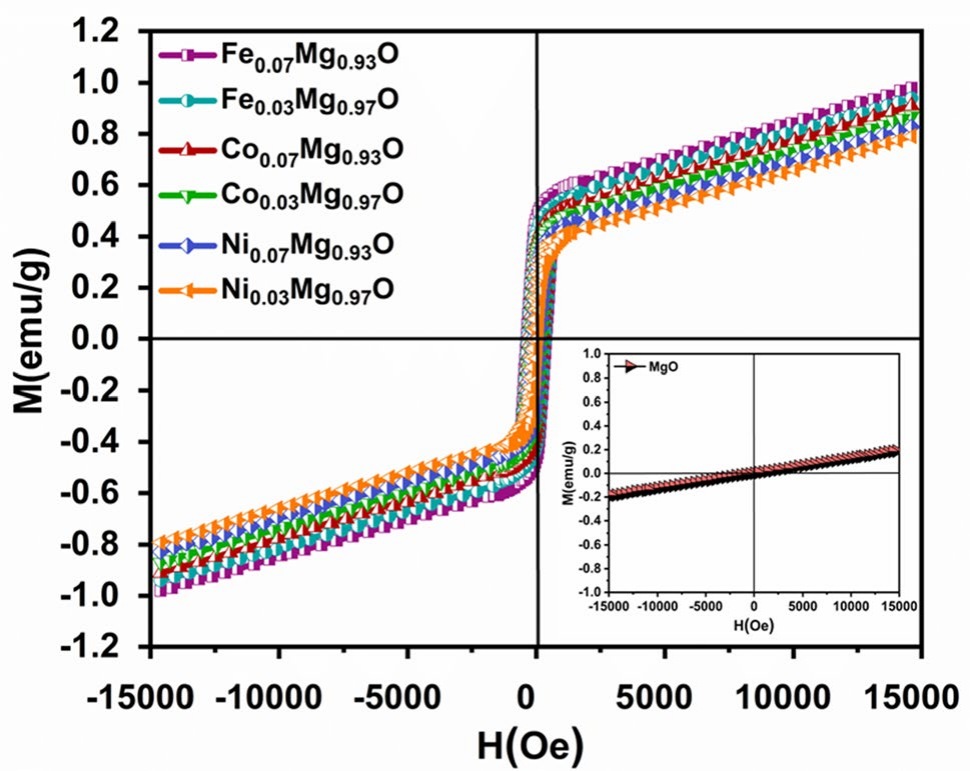
Magnetization hysteresis (M-H) curve of (Ni_x_, Co_x_, Fe_x_) Mg_1−x_O (x = 0.0, 0.03, and 0.07) nanoparticles measured at RT.

A vibrating sample magnetometer characterized the magnetic properties of divalent metal ions (Ni, Co, Fe)-doped MgO cubic nanoparticles. The origin of ferromagnetism in these samples might be due to the bound magnetic polaron (BMP) model proposed by Coey et al.^63^, which indicates that the numbers of BMPs involved overlapping polarons through oxygen vacancy defects. Magnetic properties of prepared samples were transferred from paramagnetic to ferromagnetic with dopants ions concentration. The magnetic transitions could be ascribed to the influences of the vacancy defects, either oxygen vacancies or Mg vacancies, at the surfaces of the nanocrystals and their magnetic properties. Figure 11 shows the magnetization hysteresis (M-H) curve of Ni, Co, and Fe-doped MgO nanoparticles with the different content of doping ions (0.00, 0.03, and 0.07) at room temperature. The magnetization is much larger for higher substitutions.

The significant value of saturation magnetization (Ms), remanent magnetization (Mr) and coercive field (Hc), and remanence ratio (Mr/Ms) for all synthesized samples are tabulated in Table 3. As observed, the saturation magnetization values were increased with the increase in the concentration of the dopants ions.

**Table 3:** Variations of Ms, Mr, Hc, and Mr/Ms of Ni, Co, and Fe-doped MgO nanoparticles.

Sample	M_s_ (emu/g)	M_r_ (emu/g)	H_c_ (Oe)	(M_r_/M_s_)
MgO	–	0.000	0.0000	–
Ni_0.03_ Mg_0.97_O	0.7915	0.1505	77.9754	0.1901
Ni_0.07_ Mg_0.93_O	0.9104	0.3673	262.6539	0.4034
Co_0.03_ Mg_0.97_O	0.8279	0.2309	140.9029	0.2789
Co_0.07_ Mg_0.93_O	0.9396	0.4182	322.8454	0.4450
Fe_0.03_ Mg_0.97_O	0.8701	0.3056	199.7264	0.3512
Fe_0.07_ Mg_0.93_O	0.9808	0.4692	380.3009	0.4783

**Figure 12:**
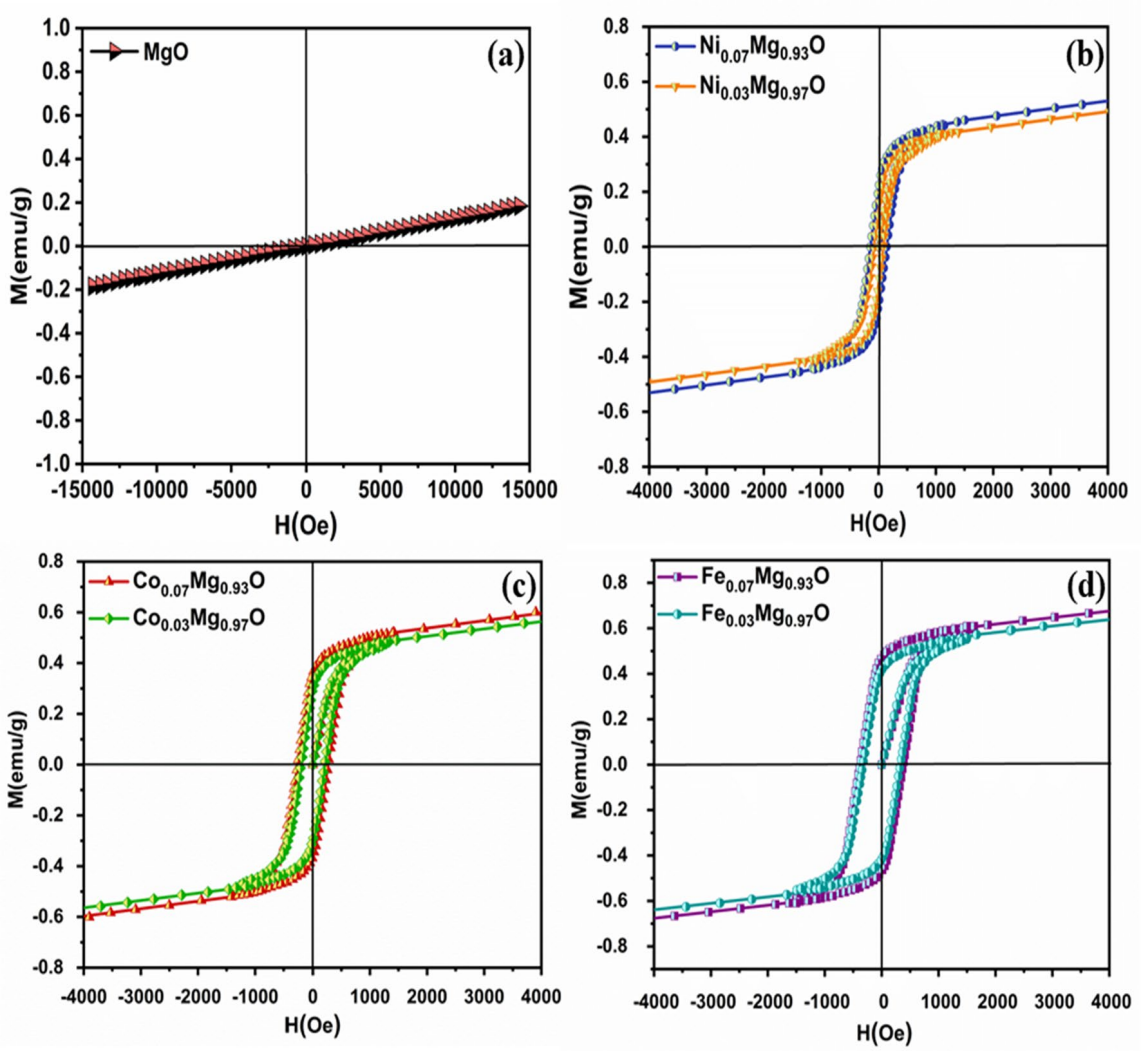
Magnetization hysteresis (M-H) curve of (**a**) pure (MgO), (**b**) 3% and 7% Ni-doped MgO, (**c**) 3% and 7% Co–MgO, and (**d**) 3% and 7% Fe-doped MgO nanoparticles measured at room temperature.

Figure 12a shows the paramagnetic behavior of MgO nanoparticles at room temperature, where Mg metal is paramagnetic in nature despite not having any unpaired electron, as well as because of alignment in nature, while doped material shows ferromagnetic nature, which might be attributed to oxygen vacancies. While VSM measurements exhibit the room temperature ferromagnetism with varying doping concentrations. The hysteresis curve shows a remnant magnetization of MgO nanoparticles is very close to zero and zero coercive field. When the substitution of divalent metal ions increases, the ferromagnetic interactions enhance. Magnetization hysteresis (M-H) loops in Ni-doped MgO at room temperature exhibited ferromagnetic behavior as shown in Fig. 12b. The magnetic properties of MgO nanoparticles changed from paramagnetic to ferromagnetic by adding doping ions into host atoms. The magnetization of Ni-doped MgO was increased with increasing the doping concentration, whereas 7% Ni-doped MgO nanoparticles has magnetic saturation higher than 3% Ni-doped MgO nanoparticles. These are attributed to induce defects/oxygen vacancy. Thus, in nanocrystalline of Ni-doped MgO nanoparticles, the oxygen vacancy would induced the ferromagnetic behavior.

Singh et al. pointed that the bond spin polarization between two Mg vacancies in MgO occurred magnetic interaction in the MgO system. Though oxygen vacancy does not induce magnetization, these vacancies can play significant roles in doped materials, as in the case of doped MgO nanoparticles^64^. Azzaza et al. reported that the pristine MgO nanoparticles exhibited two magnetization components. One is superparamagnetic. Another is diamagnetic, while bulk MgO is a diamagnetic material. They noted that density functional theory (DFT) calculations for the effect of site vacancies (O or Mg) in MgO crystal may display the paramagnetic behavior of pure MgO nanocrystal^65^. Moyses et al. confirmed that pristine MgO thin films had shown paramagnetic behavior at room temperature^66^.

In MgO nanoparticles, the magnetic properties were found to increase with increasing Co dopant ions. The arising ferromagnetic behavior in Co-doped MgO nanoparticles might be due to the ferromagnetic exchange interaction between Co^2+^ and other ions. A long-range ferromagnetic exchange Interactions could be mediated through the formation of bound magnetic polarons^67^. The presence of magnetic behavior in Co-doped MgO nanoparticles could be ascribed to the presence of intrinsic vacancies within the material. As observed in magnetic hysteresis loop Fig. 12c the magnetic behavior increased at 7% Co-doped MgO nanoparticles. Co^2+^ dopant ions lead to more defects/oxygen vacancy. At room temperature, this oxygen vacancy induced the ferromagnetic behavior in Co-doped MgO nanoparticles. Depend upon the previous analysis of ferromagnetism in MgO nanoparticles is proposed that the ferromagnetism in MgO originated from (Mg vacancies (${\text{V}}_{\text{Mg}})$ or/and oxygen vacancies (${\text{V}}_{\text{o}}$) at the surfaces of the nanocrystals. These results confirm the presence of a strong relation between ferromagnetism and ${\text{V}}_{\text{o}}$, ${\text{V}}_{\text{Mg}}$.

Figure 12d shows a hysteresis loop of Fe-doped MgO nanoparticles at room temperature. Oxygen vacancies are induced when Fe ions which are divalent, are doped in MgO lattice. A high concentration of Fe^2+^ doping ions leads to more defects/oxygen vacancy (${\text{V}}_{\text{o }})$, this ${\text{V}}_{\text{o}}$ induced ferromagnetic behavior in 3% and 7% of Fe-doped MgO nanoparticles at room temperature based on the BMP model. The magnetic behavior is increased gradually by varying Fe doping concentrations. Oxygen vacancies are mainly created. This oxygen vacancy maintains charge neutrality. In the case of Fe-doped MgO nanoparticles, the electrons are trapped through the defects when Fe^2+^ ions interact with oxygen vacancy. Their interaction causes polarization, which creates the magnetic moment, which is called BMPs. Saturation magnetization and coercive field of Fe-doped MgO nanoparticles were increased with increasing doping concentration, as tabulated in Table 3. The highest value of saturation magnetization was found for 7% of Fe-doped MgO nanoparticles compared to Ni and Co-doped MgO nanoparticles. The grain size of Fe-doped MgO nanoparticles might be one of the reasons for increased saturation magnetization and coercivity of these samples more than Ni and Co-doped MgO nanoparticles. In contrast, Fe-doped MgO nanoparticles have the smallest size in particles. The smaller grain size of the powders, which have higher surface-to-volume ratios, will result in many more surface vacancies. The presence of a high oxygen vacancy in the sample gives rise to the ferromagnetic ordering, which leads to the increment of magnetization. Phokha et al., reported the presented the room temperature ferromagnetism with a maximum magnetization of 1.60 emu/g at 0.07 Fe^2+^ ions^68^.

**Figure 13:**
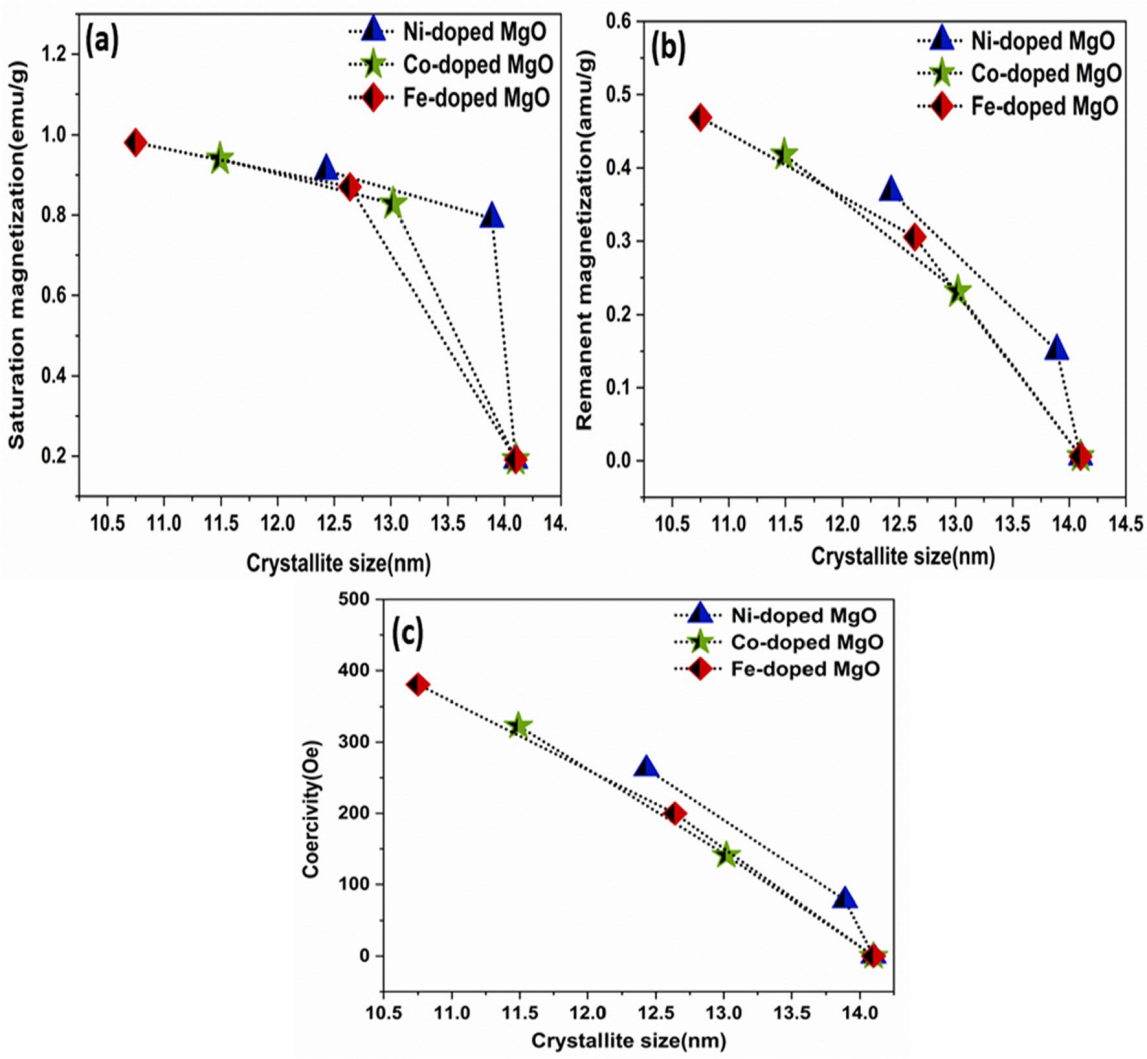
The Correlation between the crystallite size and (**a**) Ms, (**b**) Mr, (**c**) Hc of Ni_,_ Co_,_ and Fe-doped MgO nanoparticles with the various doping concentrations of 0%, 3% and 7%.

As observed, the magnetic properties of all samples were increased with the deceased in the crystallite size. The correlation between saturation magnetization (Ms), remnant magnetization (Mr), and coercive field (Hc) with the crystallite size was expressed in Fig. 13a–c.

**Figure 14:**
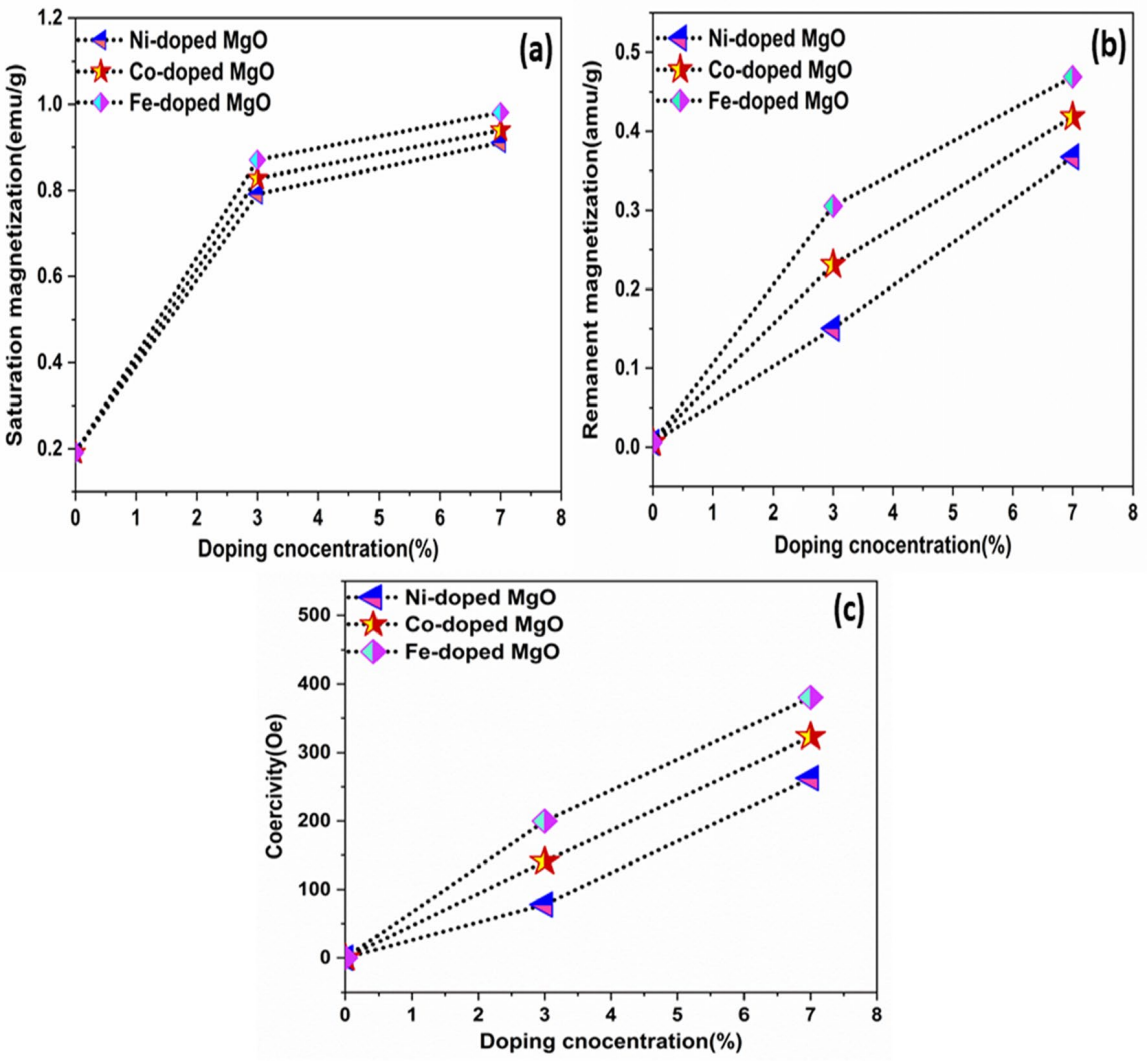
The correlation between the doping concentration and (**a**) Ms, (**b**) Mr, (**c**) Hc for Ni_,_ Co_,_ and Fe-doped MgO nanoparticles with the various doping concentrations of 0%, 3% and 7%.

The divalent metal ions (Ni, Co, and Fe) enhanced the magnetic properties of Ni, Co, and Fe-doped MgO nanoparticles by changing the concentration of dopant. Ms, Mr, and Hc were increased with increasing doping concentration. Figure 14a–c shows the correlation between doping concentration and saturation magnetization, remnant magnetization, coercivity for the used samples.

### Study and analysis the effect of antibacterial activity of transition metal oxide nanoparticles

To examine the antibacterial activity of the Ni_,_ Co, and Fe-doped MgO nanoparticles with various amounts of dopant ions of 0%, 1%, 3%, 5%, and 7%, there are two mechanisms. First, the efficiency of bacteria inhibition and bacterial cell growth of various microorganisms (negative and positive bacteria) in the presence of the considered nanoparticles was studied as following methods:

### Agar disc diffusion antibacterial activity

**Figure 15:**
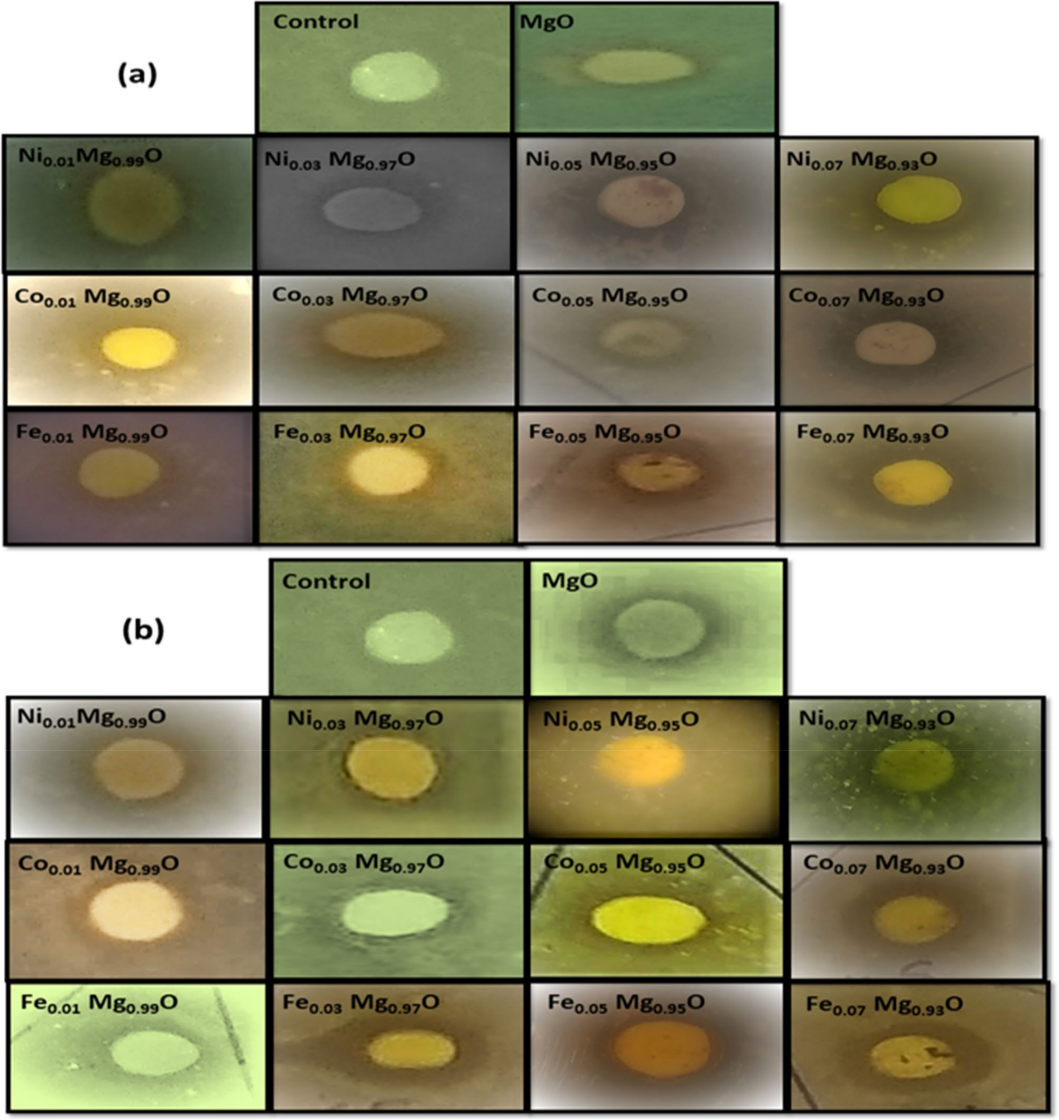
Appearances of the zone of inhibition for (**a**) 40 μg/ml of Ni, Co, and Fe-doped MgO nanoparticles. (**b**) 80 μg/ml of Ni, Co, and Fe-doped MgO nanoparticles with *E. coli.*

**Figure 16:**
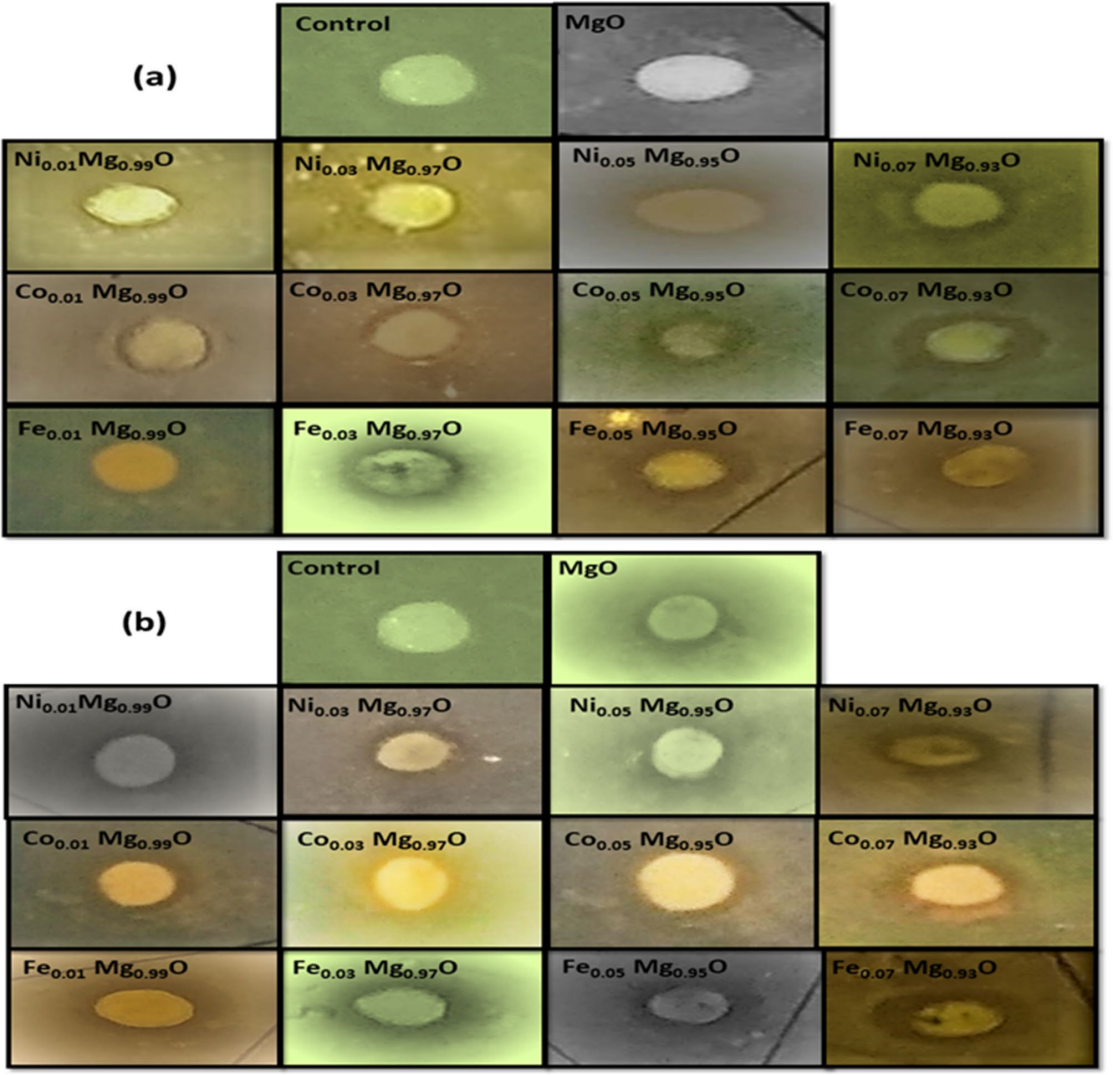
Appearances of the zone of inhibition for (**a**) 40 μg/ml of Ni, Co, and Fe-doped MgO nanoparticles. (**b**) 80 μg/ml of Ni, Co, and Fe-doped MgO nanoparticles with *S. aureus.*

*E. coli* and *S. aureus,* were used in this test. Initially, liquid and solid nutrient bacterial growth media were prepared and sterilized by autoclave at 121 °C for 60 min. All the tools (flask, test tubes, Petri dishes, and needles) used in this work were sterilized in an autoclave at 121 °C for 60 min. After cooled bacteria culture, bacteria *E. coli* and *S. aureus* was inoculated on the liquid medium (nutrient broth) and then incubated overnight at 37 °C. The serial dilutions of bacterial suspension were used to obtain 10^–4^ of the bacteria *E. coli* and *S. aureus* colony-forming units (CFU) per ml. Prepared solid medium (nutrient agar) and allowed to cool it, but not solidify in the flask. We poured into each sterilized petri dish. Allowed the nutrient agar to harden for 20 to 30 min at room temperature. 1 ml of 10^–4^ dilution of colony-forming units (CFU) was applied to the nutrient agar plates and using a glass rod for uniformly spreading it on the surface of the medium. The sterile filter paper discs were used for investigating the minimal inhibitory concentrations (MIC) of synthesized nanoparticles. The varying doses (10 μg/ml, 20 μg/ml, and 40 μg/ml) of Ni, Co, and Fe-doped MgO nanoparticles were loaded on the filter paper discs and placed over the nutrient agar surface. All assays have been done in triplicates to eliminate errors during the procedure. The processes have been done under a laminar flow hood. The solid medium (nutrient agar) without nanoparticles (untreated) and containing the same concentrations of CFU was used as blank controls under the same conditions. All the Petri dishes have been incubated overnight at 37 °C. After 24 h of incubation, the inhibition zone of the antibacterial was observed, as shown in the images of Figs. 15 and 16.

**Figure 17:**
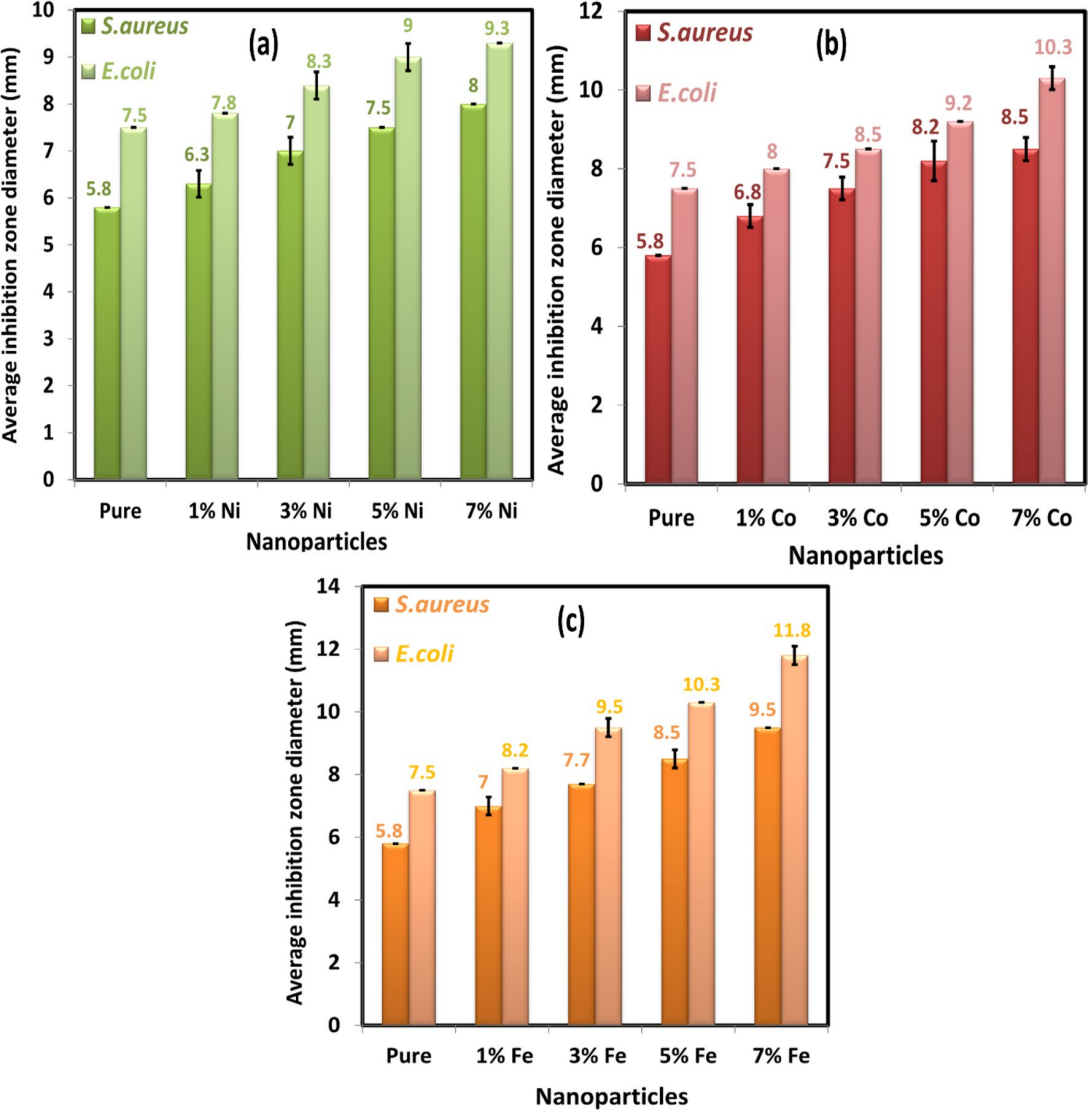
The average diameters of inhibition zone at 40 μg/ml of (**a**) Ni-doped MgO, (**b**) Co-doped MgO and (**c**) Fe-doped MgO nanoparticles with the various doping concentrations of 0%, 1%, 3%, 5%, and 7%.

**Figure 18:**
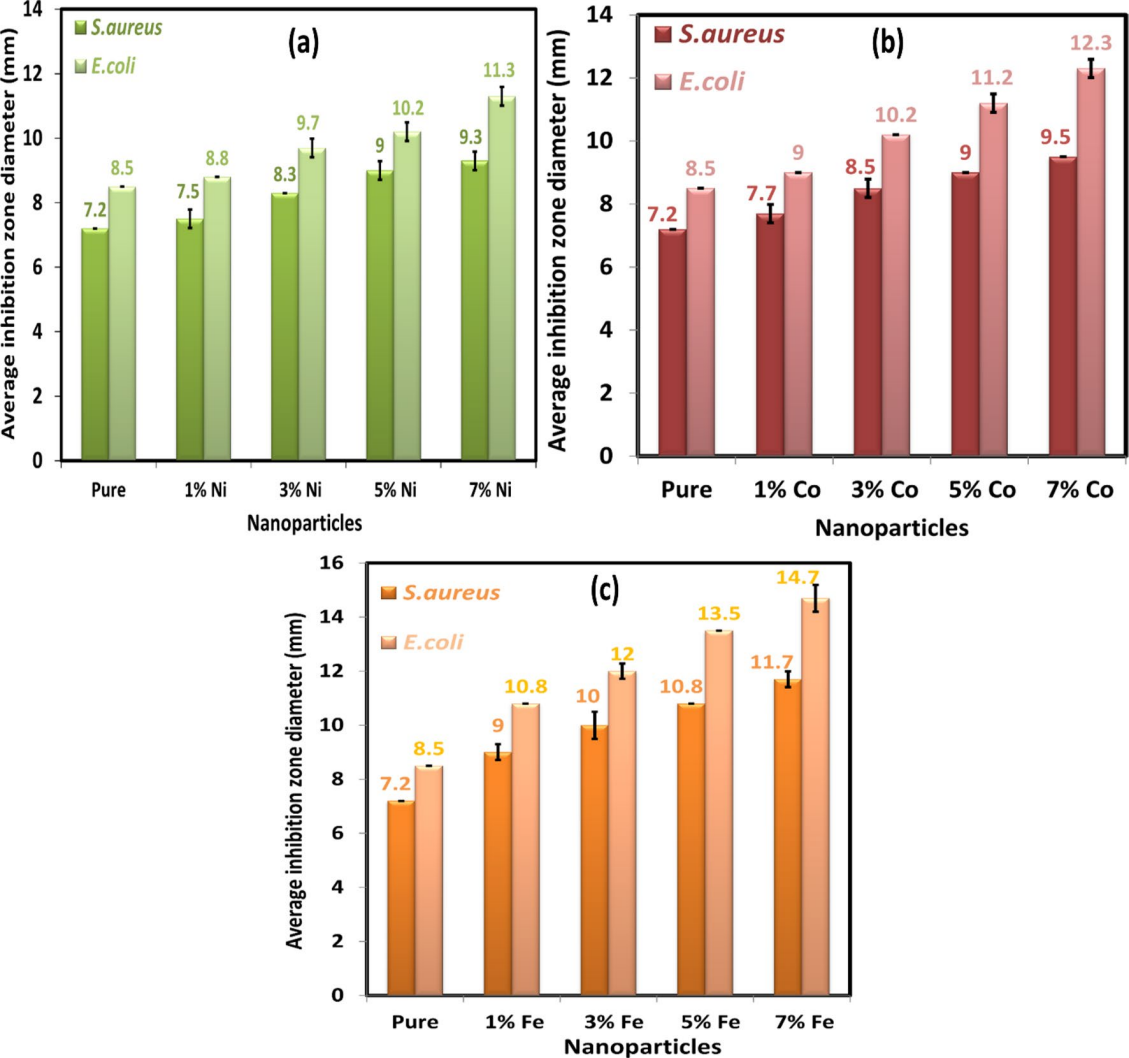
The average diameters of inhibition zone at 80 μg/ml of (**a**) Ni-doped MgO, (**b**) Co-doped MgO and (**c**) Fe-doped MgO nanoparticles with various doping concentrations of 0%, 1%, 3%, 5%, and 7%.

A scale in mm was used to measure the inhibition zone diameters, which had appeared around each filter paper disc. At doses 20 and 30 μg/ml of nanoparticle suspensions, there was no inhibition zone against *E. coli* (gram-negative) and *S. aureus* (gram-positive). Still, the inhibition zone was observed around each filter paper disk against *E. coli* and *S. aureus* at dose 40 μg/ml of nanoparticle suspensions as shown in Figs. 15 and 16. The differences measured of inhibition zone diameter around the filter paper disk might be attributed to many factors such as the amount of the nanoparticle concentrations suspension, size of nanoparticles, the type of the materials, and resistance of bacteria on antibacterial. The average inhibition zone diameter at 40 μg/ml and 80 μg/ml of Ni, Co, and Fe-doped MgO nanoparticles against *E. coli* were listed in Tables 4 and 5, respectively, as shown in Figs. 17 and 18. The values of the average diameter of the inhibition zone against *S. aureus* were listed in Tables 6 and 7 and shown in Figs. 17 and 18. The results of the effect of Ni, Co, and Fe-doped MgO nanoparticles on *E. coli* and *S. aureus* at 40 μg/ml and 80 μg/ml showed better activity with gram-negative bacteria (*E. coli*) than gram-positive bacteria (*S. aureus*). These results are possible because *S. aureus* bacteria possess a high resistance, becoming resistant to many commonly used antibiotics. As noticeable from the readings in Tables 6 and 7, the inhibition zone diameter increased by increasing the concentration of doping ions. At 7% of transition metal (Ni, Co, and Fe)-doped MgO nanoparticles, the diameters of inhibition zone bigger than the diameter of inhibition zone for 1% of transition metal (Ni, Co, and Fe)-doped MgO nanoparticles. Moreover, 7% Fe-doped MgO nanoparticles exhibit the best bactericidal effect at a 80 μg/ml concentration.

**Table 4:** The average diameter of inhibition zone of Ni, Co, and Fe-doped MgO nanoparticles at 40 μg/ml against *E. coli.*

Sample	DIZ of NPs against *E. coli* (mm)			Avg zone diam (mm) ± SD
	*a*	*b*	*c*	
Control	–	–	–	–
MgO	7.5	7.5	7.5	7.5 ± 0.00
Ni_0.01_ Mg_0.99_O	8.0	8.0	7.5	7.8 ± 0.289
Ni_0.03_ Mg_0.97_O	8.5	8.5	8.0	8.3 ± 0.289
Ni_0.05_ Mg_0.95_O	9.0	9.0	9.0	9.0 ± 0.00
Ni_0.07_ Mg_0.93_O	9.0	9.5	9.5	9.3 ± 0.289
Co_0.01_ Mg_0.99_O	8.0	8.0	8.0	8.0 ± 0.00
Co_0.03_ Mg_0.98_O	8.5	8.5	8.5	8.5 ± 0.00
Co_0.05_ Mg_0.95_O	9.0	9.5	9.0	9.2 ± 0.289
Co_0.07_ Mg_0.93_O	10.0	10.5	10.5	10.3 ± 0.289
Fe_0.01_ Mg_0.99_O	8.0	8.5	8.0	8.2 ± 0.289
Fe_0.03_ Mg_0.98_O	9.5	9.5	9.5	9.5 ± 0.00
Fe_0.05_ Mg_0.95_O	10.0	10.5	10.5	10.3 ± 0.289
Fe_0.07_ Mg_0.93_O	11.5	12.0	12.0	11.8 ± 0.289

**Table 5:** The average diameter of inhibition zone of Ni, Co, and Fe-MgO nanoparticles at 80 μg/ml against *E. coli*.

Sample	DIZ of NPs against *E. coli* (mm)			Avg zone diam (mm) ± SD
	*a*	*b*	*c*	
Control	–	–	–	–
MgO	8.5	8.5	8.5	8.5 ± 0.00
Ni_0.01_ Mg_0.99_O	8.5	9.0	9.0	8.8 ± 0.289
Ni_0.03_ Mg_0.97_O	9.5	9.5	10.0	9.7 ± 0.289
Ni_0.05_ Mg_0.95_O	10.0	10.5	10.0	10.2 ± 0.289
Ni_0.07_ Mg_0.93_O	11.0	11.5	11.5	11.3 ± 0.289
Co_0.01_ Mg_0.99_O	9.0	9.0	9.0	9.0 ± 0.00
Co_0.03_ Mg_0.98_O	10.0	10.0	10.5	10.2 ± 0.289
Co_0.05_ Mg_0.95_O	11.0	11.5	11.0	11.2 ± 0.289
Co_0.07_ Mg_0.93_O	12.0	12.5	12.5	12.3 ± 0.289
Fe_0.01_ Mg_0.99_O	10.5	11.0	11.0	10.8 ± 0.289
Fe_0.03_ Mg_0.98_O	12.0	12.0	12.0	12.0 ± 0.00
Fe_0.05_ Mg_0.95_O	13.0	13.5	14.0	13.5 ± 0.50
Fe_0.07_ Mg_0.93_O	14.5	14.5	15.0	14.7 ± 0.289

**Table 6:** The average diameter of inhibition zone of Ni, Co, and Fe-MgO nanoparticles at 40 μg/ml against *S. aureus*.

Sample	DIZ of NPs against *S. aureus* (mm)			Avg zone diam (mm) ± SD
	*a*	*b*	*c*	
Control	–	–	–	–
MgO	6.0	6.0	5.5	5.8 ± 0.289
Ni_0.01_ Mg_0.99_O	6.0	6.5	6.5	6.3 ± 0.289
Ni_0.03_ Mg_0.97_O	7.0	7.0	7.0	7.0 ± 0.00
Ni_0.05_ Mg_0.95_O	7.5	7.5	7.5	7.5 ± 0.00
Ni_0.07_ Mg_0.93_O	8.0	8.0	8.0	8.0 ± 0.00
Co_0.01_ Mg_0.99_O	6.5	7.0	7.0	6.8 ± 0.289
Co_0.03_ Mg_0.98_O	7.5	7.0	8	7.5 ± 0.50
Co_0.05_ Mg_0.95_O	8.0	8.0	8.5	8.2 ± 0.289
Co_0.07_ Mg_0.93_O	8.5	8.5	8.5	8.5 ± 0.00
Fe_0.01_ Mg_0.99_O	7.0	7.0	7.0	7.0 ± 0.00
Fe_0.03_ Mg_0.98_O	7.5	7.5	8.0	7.7 ± 0.289
Fe_0.05_ Mg_0.95_O	8.5	8.5	8.5	8.5 ± 0.00
Fe_0.07_ Mg_0.93_O	9.5	9.5	9.5	9.5 ± 0.00

**Table 7:** The average diameter of inhibition zone of Ni, Co, and Fe-MgO nanoparticles at 80 μg/ml against *S. aureus*.

S. no	Sample	DIZ of NPs against *S. aureus* (mm)			Avg zone diam (mm) ± SD
		*a*	*b*	*c*	
1	Control	–	–	–	–
2	MgO	7.5	7.0	7.0	7.2 ± 0.289
3	Ni_0.01_ Mg_0.99_O	7.5	7.5	7.5	7.5 ± 0.00
4	Ni_0.03_ Mg_0.97_O	8.0	8.5	8.5	8.3 ± 0.289
5	Ni_0.05_ Mg_0.95_O	8.5	9.0	9.0	8.8 ± 0.289
6	Ni_0.07_ Mg_0.93_O	9.5	9.0	9.5	9.3 ± 0.289
7	Co_0.01_ Mg_0.99_O	7.5	7.5	8.0	7.7 ± 0.289
8	Co_0.03_ Mg_0.98_O	8.5	8.5	8.5	8.5 ± 0.00
9	Co_0.05_ Mg_0.95_O	9.0	9.0	9.0	9.0 ± 0.00
10	Co_0.07_ Mg_0.93_O	9.5	9.5	9.5	9.5 ± 0.00
11	Fe_0.01_ Mg_0.99_O	8.5	9.0	9.5	9.0 ± 0.50
12	Fe_0.03_ Mg_0.98_O	10.0	10.0	10.0	10.0 ± 0.00
13	Fe_0.05_ Mg_0.95_O	10.5	11.0	11.0	10.8 ± 0.289
14	Fe_0.07_ Mg_0.93_O	11.5	11.5	12.0	11.7 ± 0.00

### Pour plate technique

The pour plate technique was used to count the total number of colony-forming bacteria present in the liquid media. We used the mixed samples of nanoparticles colloidal suspension and bacterial suspension in this technique. First, liquid and solid nutrient bacterial growth media were prepared and sterilized for 60 min at 121 °C in an autoclave. After using autoclave, the nutrient broth medium was kept for a few minutes under room temperature till it became cool. Then it was inoculated by *E. coli* and *S. aureus* in separate test tubes and incubated for 24 h at 37 °C. The serial dilution was prepared up to 10^–4^ of CFU to reduce bacterial suspension concentration. The bacterial suspensions were inoculated using nanoparticles colloidal suspensions of Ni-doped MgO, Co-doped MgO, and Fe-doped MgO nanoparticles. 1 ml of each mixed suspension of the samples for each test tube was poured into petri dishes by sterilizing pipette, and then molten nutrient agar was poured on it, equilibrated to a temperature of about 48 °C. The lid of petri dishes was replaced directly. The bacteria must achieve uniform distribution in the agar plates, so the plate should gently rotate in a circular motion. The agar is allowed to solidify for about 30 min, and then the plates are inverted for incubation. One of the Petri dishes was kept as the control without treatment to compare the treated and untreated samples. In this work, the petri dish was plated in triplicate for more accuracy in the growing bacterial colonies number (treated and untreated). All the samples were incubated overnight at 37 °C. After 24 h of incubation, the growth of bacterial colonies was counted and calculated the average in the triplicate. Lastly, to evaluate the antimicrobial properties of the samples, a comparison has been made between the numbers of growing bacterial colonies treated with the number of growing bacterial colonies that were untreated by nanoparticles.

**Figure 19:**
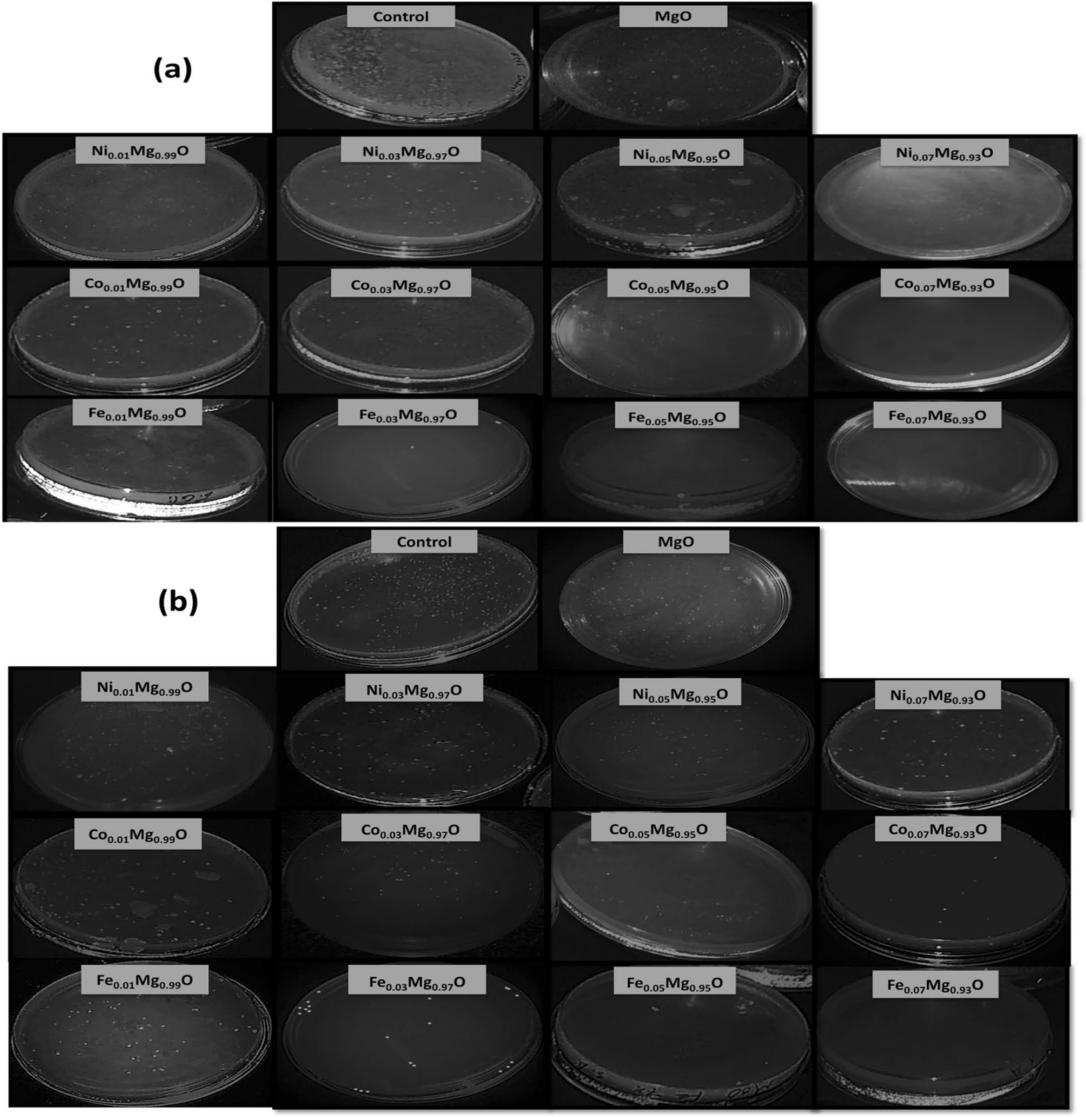
Representative photographs of recultivated bacteria colonies of (**a**) *E. coli* and (**b**) *S. aureus* on nutrient agar culture plates treated with 80 μg/ml of different nanoparticles.

Figure 19a,b showed the effective of pure MgO and Ni, Co, and Fe-doped MgO nanoparticles with dopant concentrations of 1, 3, 5, and 7% against *E. coli* and *S. aureus* bacterium. Clearly, all undoped and Ni, Co, and Fe-doped MgO nanoparticles specimens show good antibacterial activity as compared with the blank control group. Noted that 7% Fe-doped MgO NPs exhibited excellent bacterial growth inhibition effect than pure MgO nanoparticles though there existed several discernable bacterial strains. The number of viable colonies and the efficiency of inhibiting *E. coli* (gram-negative) and *S. aureus* (gram-positive) was tabulated in Tables 8 and 9. The Colony Forming Units/ml was decreased with the increase of the concentration of transition metal (Ni, Co, and Fe), as we observed in the images of Fig. 19a,b. The results revealed that the transition metal (Ni, Co, and Fe)-doped MgO nanoparticles having suitable activities with the *E. coli* more than *S. aureus*, in which *S. aureus* bacteria showed a stronger resistance to MgO and transition metal (Ni, Co, and Fe)-doped MgO nanoparticles as compared to *E. coli bacteria.* The difference among them is that the transition metal Fe-doped MgO nanoparticles possess a higher activity with the *E. coli* (gram-negative) and *S. aureus* (gram-positive) more than the transition metal (Ni and Co)-doped MgO nanoparticles.

**Table 8:** The efficiency of inhibition of Ni, Co, and Fe-doped MgO nanoparticles at 80 μg/ml against *E. coli.*

S. no	Sample	*E. coli* (CFU/ml) × 10^4^	Efficiency of inhibition (%)
1	Control	222	0.00
2	MgO	172	22.52
3	Ni_0.01_ Mg_0.99_O	68	69.37
4	Ni_0.03_ Mg_0.97_O	52	76.58
5	Ni_0.05_ Mg_0.95_O	25	88.74
6	Ni_0.07_ Mg_0.93_O	9	95.95
7	Co_0.01_ Mg_0.99_O	44	80.18
8	Co_0.03_ Mg_0.98_O	38	82.88
9	Co_0.05_ Mg_0.95_O	10	95.50
10	Co_0.07_ Mg_0.93_O	0	100.00
11	Fe_0.01_ Mg_0.99_O	34	84.68
12	Fe_0.03_ Mg_0.98_O	5	97.75
13	Fe_0.05_ Mg_0.95_O	4	98.20
14	Fe_0.07_ Mg_0.93_O	0	100.00

**Table 9:** The efficiency of inhibition of Ni, Co, and Fe-doped MgO nanoparticles at 80 μg/ml against *S. aureus.*

S. no	Sample	*S. aureus* (CFU/ml) × 10^4^	Efficiency of inhibition (%)
1	Control	254	0.00
2	MgO	200	21.26
3	Ni_0.01_ Mg_0.99_O	103	59.45
4	Ni_0.03_ Mg_0.97_O	93	63.39
5	Ni_0.05_ Mg_0.95_O	67	73.62
6	Ni_0.07_ Mg_0.93_O	35	86.22
7	Co_0.01_ Mg_0.99_O	92	63.78
8	Co_0.03_ Mg_0.98_O	49	80.71
9	Co_0.05_ Mg_0.95_O	23	90.94
10	Co_0.07_ Mg_0.93_O	13	94.88
11	Fe_0.01_ Mg_0.99_O	61	75.98
12	Fe_0.03_ Mg_0.98_O	22	91.34
13	Fe_0.05_ Mg_0.95_O	6	97.64
14	Fe_0.07_ Mg_0.93_O	0	100.00

Most nanomaterials have antibacterial activity ascribed to various mechanisms, such as a strong reactive oxygen species (ROS), causing DNA damage and bacterial cell membrane^69^. Thus, the Ni, Co, Fe-doped MgO nanoparticles can interact with thiol groups of essential bacteria enzymes leading to their inactivation and cell death^50^. In another potential mode of mechanisms, the results of the antibacterial action of Ni, Co, Fe-doped MgO nanoparticles can be explained through the particle facilitated transport which the smaller size of metal oxide nanoparticles flood into the gram-negative bacteria cell wall. The cell wall of gram-negative bacteria consists of a single peptidoglycan layer (peptidoglycan is only a few nanometers thick) surrounded by a unique outer membrane. In contrast, the gram-positive bacteria cell wall contains many peptidoglycans layers (peptidoglycan is 30–100 nm thick), so gram-positive bacteria try to resist Ni, Co, Fe-doped MgO nanoparticles by their cell wall. By the outer bacterial membrane, the bacterial cell wall was more exposed to nanoparticles. Through the surface of microorganisms, the interaction between metal oxide nanoparticles and the bacterial cell membrane is occurred due to the uniquely high surface to volume ratio of metal oxide nanoparticles. This results in the aggregation of nanoparticles on the cell surface, leading to the bacterium’s death.

Furthermore, many studies have signified that MgO nanoparticles have dosage-dependent antibacterial activity due to the particle size-dependent antibacterial effects. For example, jin and He reported that higher concentrations of MgO nanoparticles cause more significant bacterial inactivation^70^. In addition, Sawai proved that increasing MgO concentration leads to increasing the activity of MgO nanoparticles against *E. coli*^71^*.*

**Figure 20:**
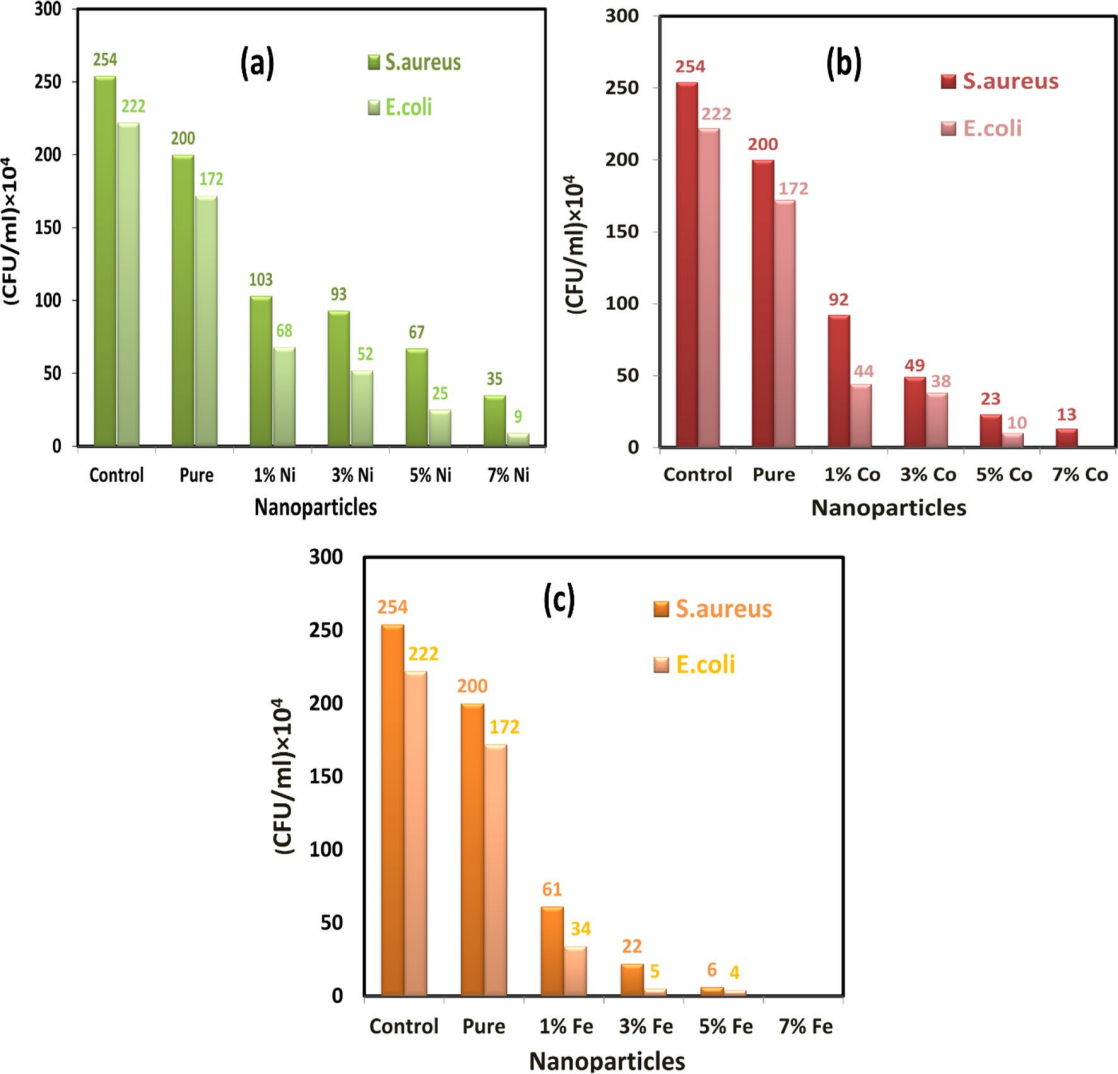
Number of *E. coli* bacterial colonies and *S. aureus* bacterial colonies as a function of (**a**) Ni-doped MgO, (**b**) Co-doped MgO and (**c**) Fe-doped MgO nanoparticles with various doping concentrations of 0%, 1%, 3%, 5%, and 7%.

**Figure 21:**
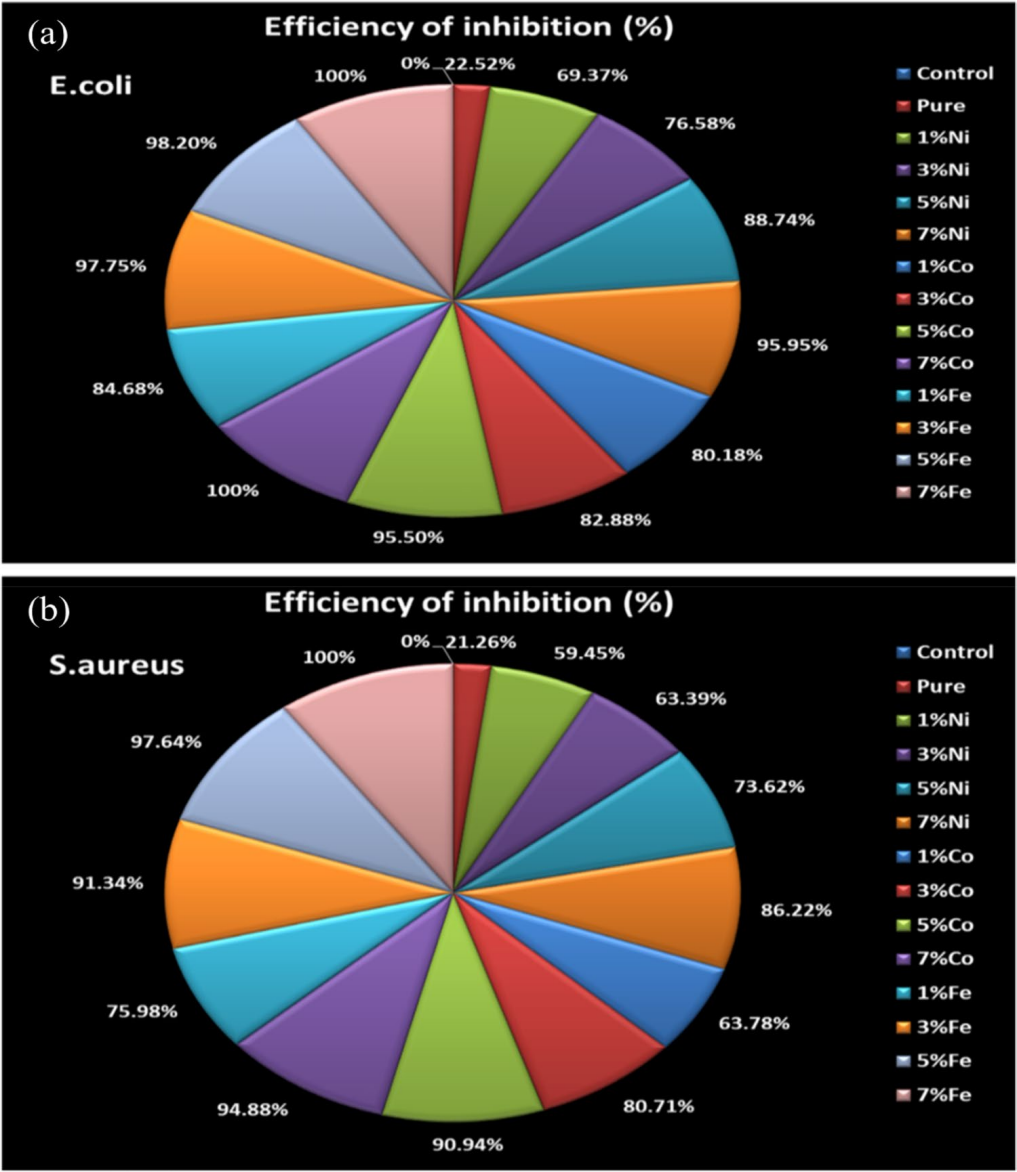
The efficiency of inhibition of nanoparticles at 80 μg/ml against (**a**) *E. coli* and (**b**) *S. aureus.*

As observed in Fig. 20a–c, the CFU of *E. coli* and *S. aureus* was decreased with the increasing concentrations of transition metal (Ni, Co, and Fe). Figure 20a–c showed that 7% of the transition metal Fe-doped MgO nanoparticles having a good effect on both classes of bacteria gram-negative (*E. coli*) and gram-positive (*S. aureus*), with a comparison between all of the different concentrations of the dopant transition metal as we have seen that 7% of transition metal Fe-doped MgO nanoparticles possess good activity against the bacteria *E. coli* (gram-negative) more than *S. aureus* (gram-positive). Bacterial growth of *E. coli* and *S. aureus* were inhibited in the presence of the prepared particles at a concentration of 80 μg/ml. Figure 21 for (a) *E. coli* (b) *S. aureus* display the percentage of the inhibition efficiency of Ni, Co, Fe-doped MgO nanoparticles at 80 μg/ml. The maximum growth inhibition of bacteria was recorded with the 7% Co and Fe-doped MgO nanoparticles, whereas the inhibition efficiency of 7% Co and Fe-doped MgO nanoparticles were about 100% for *E. coli* and 7% Fe-doped MgO nanoparticles was 100% for *S. aureus* as shown in Fig. 21a,b. In the case of *E. coli*, the bacterial growth was inhibited by 95.95% at 80 μg/ml of 7%Ni MgO nanoparticles, while the complete growth inhibition was achieved with 7% Co, and 7% Fe-doped MgO nanoparticles. In contrast, experiments with the bacterium *S. aureus* achieved the complete inhibition of bacterial growth at 80 μg/ml of 7% Fe-doped MgO, while bacterial growth was inhibited by 94.88% in the presence of 7% Co-doped MgO, as it was also inhibited by 86.22% with 7%Ni-doped MgO nanoparticles at a concentration of 80 μg/ml.

### Surface morphology of bacterial cells before and after treatment with prepared nanoparticles

**Figure 22:**
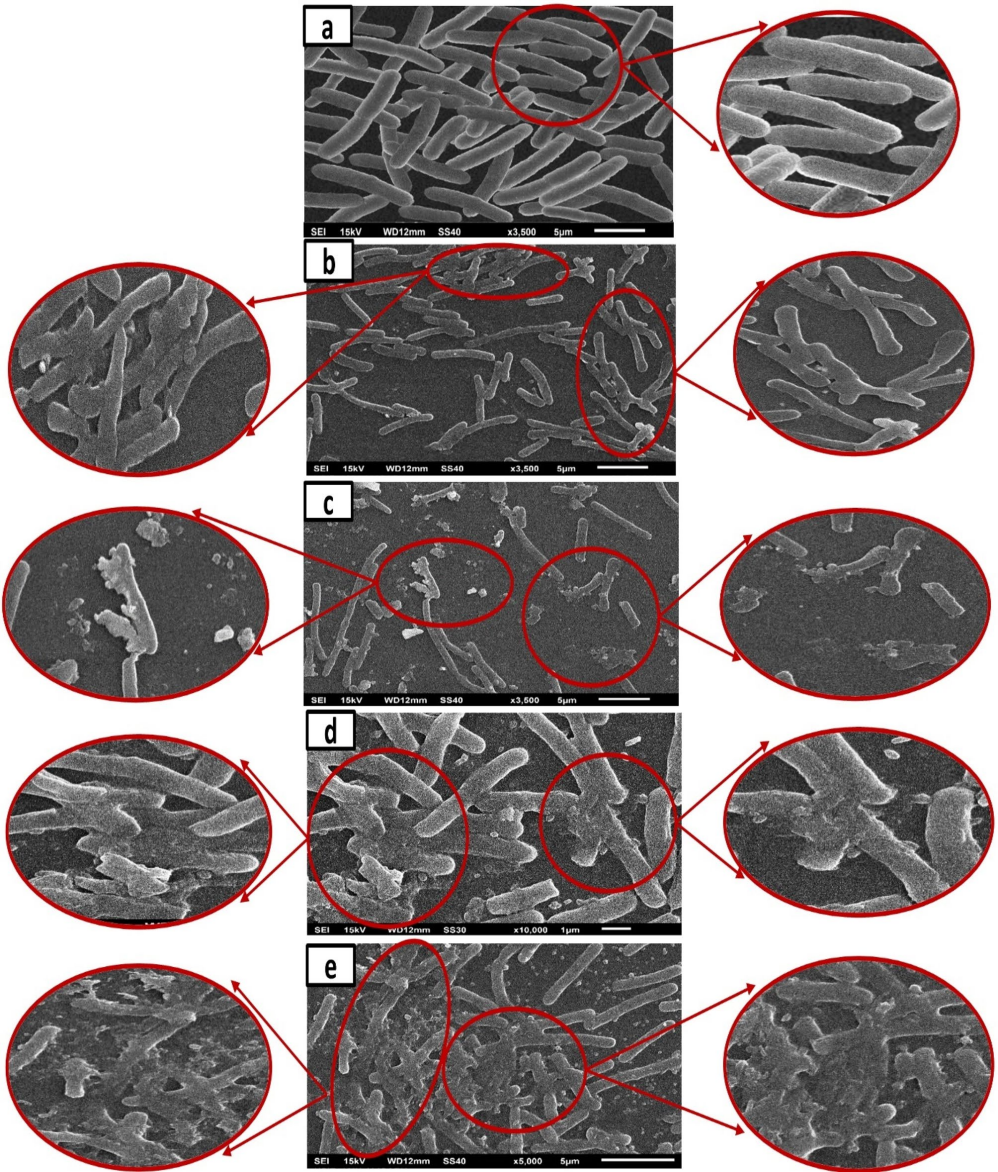
Scanning electron microscopy (SEM) images of *E. coli* cells of (**a**) untreated, (**b**) treated with MgO nanoparticles, (**c**) treated with 5% of Ni-doped MgO nanoparticles, (**d**) treated with 5% of Co-doped MgO nanoparticles, and (**e**) treated with 5% of Fe-doped MgO nanoparticles. Red circles indicate membrane regions of *E. coli* damaged by nanoparticles.

Antibacterial efficiency of pure MgO nanoparticles and 5% of Ni, Co, and Fe-doped MgO nanoparticles were also evaluated by detecting morphological changes in the *E. coli* cells before and after exposure to the nanoparticles. SEM was used to illustrate the interaction between bacterial cells and 5% of Ni, Co, and Fe-doped MgO nanoparticles. Figure 22 displays the images of *E. coli* cells either untreated (control) or treated with nanoparticles. Figure 22a shows the control cells of *E. coli*, which clearly had a rod-like shape with smooth intact surfaces. In contrast, the treated *E. coli* cells show the disintegration of the cell wall, as shown in Fig. 22b–e, the extensive membrane damage was observed for *E. coli* cells treated with 5% of Fe-doped MgO nanoparticles more than pure MgO, and 5% of Ni, Co doped MgO nanoparticles.

**Figure 23:**
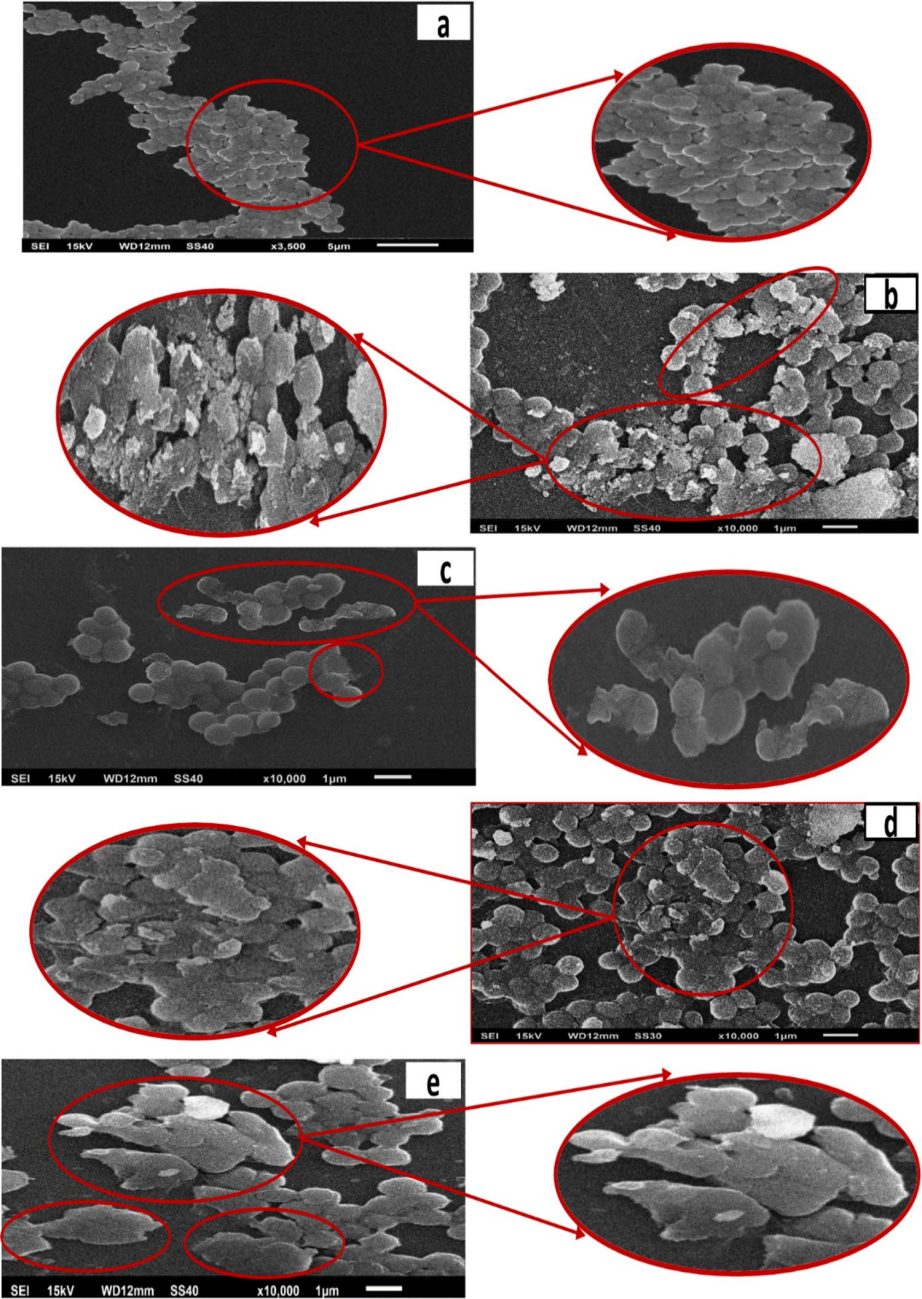
Scanning electron microscopy (SEM) images of *S. aureus* cells. (**a**) Untreated, (**b**) treated with 5% of Ni-doped MgO nanoparticles, (**c**) treated with 5% of Co-doped MgO nanoparticles, and (**d**) treated with 5% of Fe-doped MgO nanoparticles. Red circles indicate membrane regions of *S. aureus* damaged by nanoparticles.

In Fig. 23, SEM images demonstrated the effects of nanoparticles on the cell wall of *S. aureus*. SEM analysis sustains the hypothesis of membrane disruption. Figure 23a shows the spherical shape of cocci cells with smooth intact surfaces of control *S. aureus* cells, whereas *S. aureus* cells treated with pure MgO nanoparticles and 5% of Ni, Co, and Fe-doped MgO nanoparticles illustrated irregularly shaped (wrinkled/distorted morphology) with extensively damaged bacterial morphologies and lysed cells, as shown in Fig. 23b–e. It is also worth noting that 5% of Fe-doped MgO nanoparticles was able to destroy bacterial cell of *S. aureus* far more affectively as compared to pure MgO nanoparticles as well as 5% of Ni, Co-doped MgO nanoparticles. Therefore, all the findings of damaged bacterial cells of *E. coli* and *S. aureus* by pure and 5% of Ni, Co, and Fe-doped MgO nanoparticles were effective in penetrating the cells and disrupting their vital functions such as cell metabolism, cell division, DNA replication, etc. with the expulsion of cellular contents. The interaction between the nanoparticles and the cell wall of bacteria was changed due to doping of Ni, Co, and Fe. The bacterial growth of *E. coli* and *S. aureus* was more commendably affected by Co, and Fe-doped MgO nanostructures compared with Ni-doped MgO nanoparticles. The difference in the antibacterial activity of Ni, Co, and Fe-doped MgO nanostructures against Gram-negative and Gram-positive bacterial strains may be due to the difference in the cell wall structure of those pathogen bacteria. Earlier studies showed that various bacterial strains had considerably different infectivity and tolerance levels towards the other agents including antibiotics^72^. Also differences in the antibacterial activity might be due to the particle size or differences in particles dissolution behavior^73^. The antibacterial efficiency of pure MgO and Ni, Co, and Fe-doped MgO NPs is mainly dependent on the increased levels of reactive oxygen species (ROS). This is primarily due to the higher surface area which causes an increase in oxygen vacancies as well as the diffusion ability of the reactant molecules inside the nanoparticles. The transition metal-ions doping is responsible for releasing cell content outside the cell membrane through the promoting reactive oxygen species (ROS) generation^74–78^. The oxidative damage of bacterial cells occurs due to the formation of ROS production (including superoxide anions, hydroxyl radicals, hydrogen radicals), which are ultimately responsible for the dismantling of cells of bacteria leading to cell death^79^.

Furthermore, the addition of the nanoparticles on the surface of the bacteria destroys cellular function and disorganization of the cell membranes. Raj et al.^80^ reported that pure and Mg-doped ZnO nanostructures inhibited the growth of both bacteria (*E. coli* and *S. aureus*), and the zone of inhibition is proportional with the content of Mg doping in ZnO host lattice. Ohira et al.^81^ demonstrated that the antibacterial activity was enhanced with increasing content of Zn doping in MgO lattice. They pointed out that the antibacterial activity toward *S. aureus* was greater than that toward *E. coli* bacterium. Lv et al.^82^ proved that the Mg, Zn and Ce-doped CuO nanoparticles exhibited good antibacterial activity against the *E. coli* and *S. aureus* bacterium, and among them 5% Mg, 3% Zn, and 5% Ce-doped CuO nanoparticles showed the best bactericidal effect at a concentration of 0.05 mg/ml.

In the present work, we proved that the most potent antibacterial activity was achieved by 100% at a very low concentration of 80 μg/ml with 7% of Co, and Fe-doped MgO nanoparticles toward bacteria *E. coli* and 7% of Fe-doped MgO toward *S. aureus* bacterium. These types of nanoparticles achieved the complete inhibition of bacterial growth. The improved antibacterial activity of the doped MgO nanoparticles might be attributed to the synergetic effect of ROS generation and the inactivation in the bacterial cells by the binding of the Ni^2+^, Co^2+^, and Fe^2+^ ions-doped MgO to the bacterial cell surface.

## Summary and future remarks

In this work, the different concentrations of Ni, Co, and Fe ions were doped with MgO nanoparticles via sol–gel routes. The XRD analysis confirmed the presence of the cubic structure of Ni, Co, and Fe-doped MgO nanoparticles, which is indicated by the substitution of Ni, Co, and Fe into the MgO lattice. UV–Vis measurements suggested that the band-gap increased with increasing dopant ions. FESEM showed that all samples have spherical morphology. The particles size of the samples was calculated by the FE-SEM distribution curve, which was decreased with increasing doping concentration. PL studies pointed out the presence of oxygen vacancy defects in all the samples. Magnetic behavior of Ni, Co, and Fe-doped MgO system varied with Ni, Co, and Fe content. The paramagnetic behaviour changed into ferromagnetic for the doped samples. The magnetic character was increased with the addition of Ni, Co, and Fe ions. When Ni, Co, and Fe ions are divalent, doped in MgO lattice, oxygen vacancies are mainly created. The creation of oxygen vacancies results in enhanced saturation magnetization or ferromagnetism ordering. The magnetic nature of samples makes them suitable for a broader range of biomedical applications such as hyperthermia therapy of tumors, magnetic resonance imaging contrast agents, drug delivery, and others*.* The antibacterial activity of Ni, Co, and Fe-doped MgO showed more effectiveness in inhibiting the growth of bacteria *E. coli* as compared to bacteria *S. aureus*. Ni, Co, and Fe-doped MgO nanoparticles at 80 μg/ml concentration showed a remarkable maximum inhibition zone against *E. coli* and *S. aureus*, while the minimum inhibition zone was clearly remarked at 40 μg/ml.

Furthermore, the effect of Ni, Co, and Fe-doped MgO nanoparticles at the concentration of 80 μg/ml confirmed that Fe-doped MgO was more efficient to restrict the bacterial cell growth than Ni, Co-doped MgO nanoparticles. This work concluded that antibacterial tests achieved the efficiency of prepared nanoparticles of 100% with 7% (Co/Fe)-doped MgO against bacteria *E. coli*, and 100% with 7% Fe-doped MgO against bacteria *S. aureus*. As our findings cleared, with the smallest nanoparticles of 7% Fe-doped MgO, the bacterial growth was utterly inhibited, which might be due to the ease of penetration in the bacterial cell membrane. This proves that the nanoparticle size correlates with the growth inhibition of the bacterium. SEM images confirmed that the treated bacterial cells of *E. coli* and *S. aureus* were significantly changed and showed significant damage in the bacterial cells.

Based on these results, we expect that these materials will open a new window for the researchers to give bright insights and new uses of these materials, especially in biomedical applications, such as involving in biomedical and pharmaceutical industries and controlling the use of antibiotics. In addition, due to the magnetic nature of these materials, they can act as topical drug delivery systems. So, the research perspective of these materials is critical to explore.

## Data Availability

All data generated or analysed during this study are included in this published article.
